# Groundwater-dependent ecosystem map exposes global dryland protection needs

**DOI:** 10.1038/s41586-024-07702-8

**Published:** 2024-07-17

**Authors:** Melissa M. Rohde, Christine M. Albano, Xander Huggins, Kirk R. Klausmeyer, Charles Morton, Ali Sharman, Esha Zaveri, Laurel Saito, Zach Freed, Jeanette K. Howard, Nancy Job, Holly Richter, Kristina Toderich, Aude-Sophie Rodella, Tom Gleeson, Justin Huntington, Hrishikesh A. Chandanpurkar, Adam J. Purdy, James S. Famiglietti, Michael Bliss Singer, Dar A. Roberts, Kelly Caylor, John C. Stella

**Affiliations:** 1 California Water Program, The Nature Conservancy, San Francisco, CA USA; 2 State University of New York, College of Environmental Science and Forestry, Syracuse, NY USA; 3 Rohde Environmental Consulting, LLC, Seattle, WA USA; 4 Division of Hydrologic Sciences, Desert Research Institute, Reno, NV USA; 5 Department of Civil Engineering, University of Victoria, Victoria, British Columbia Canada; 6 Global Institute for Water Security, University of Saskatchewan, Saskatoon, Saskatchewan Canada; 7 International Institute for Applied Systems Analysis, Laxenburg, Austria; 8 The World Bank, Washington, DC USA; 9 The Nature Conservancy, Reno, NV USA; 10 Oregon Sustainable Water Program, The Nature Conservancy, Bend, OR USA; 11 Freshwater Biodiversity Programme, South African National Biodiversity Institute, Cape Town, South Africa; 12 The Nature Conservancy, Hereford, AZ USA; 13 Resilient Rivers LLC, Hereford, AZ USA; 14 International Platform for Dryland Research and Education, Tottori University, Tottori, Japan; 15 Graduate School of Bioresources, Mie University, Tsu, Japan; 16 School of Earth and Ocean Sciences, University of Victoria, Victoria, British Columbia Canada; 17 Center for Sustainability, Environment, and Climate Change, FLAME University, Pune, India; 18 California State University, Monterey Bay, Seaside, CA USA; 19 School of Environment and Sustainability, University of Saskatchewan, Saskatoon, Saskatchewan Canada; 20 School of Sustainability, Arizona State University, Tempe, AZ USA; 21 School of Earth and Environmental Sciences, Cardiff University, Cardiff, UK; 22 Water Research Institute, Cardiff University, Cardiff, UK; 23 Earth Research Institute, University of California, Santa Barbara, CA USA; 24 Department of Geography, University of California, Santa Barbara, CA USA; 25 Bren School of Environmental Science and Management, University of California Santa Barbara (UCSB), Santa Barbara, CA USA

**Keywords:** Environmental impact, Freshwater ecology, Hydrology, Conservation biology, Water resources

## Abstract

Groundwater is the most ubiquitous source of liquid freshwater globally, yet its role in supporting diverse ecosystems is rarely acknowledged^1,2^. However, the location and extent of groundwater-dependent ecosystems (GDEs) are unknown in many geographies, and protection measures are lacking^1,3^. Here, we map GDEs at high-resolution (roughly 30 m) and find them present on more than one-third of global drylands analysed, including important global biodiversity hotspots^4^. GDEs are more extensive and contiguous in landscapes dominated by pastoralism with lower rates of groundwater depletion, suggesting that many GDEs are likely to have already been lost due to water and land use practices. Nevertheless, 53% of GDEs exist within regions showing declining groundwater trends, which highlights the urgent need to protect GDEs from the threat of groundwater depletion. However, we found that only 21% of GDEs exist on protected lands or in jurisdictions with sustainable groundwater management policies, invoking a call to action to protect these vital ecosystems. Furthermore, we examine the linkage of GDEs with cultural and socio-economic factors in the Greater Sahel region, where GDEs play an essential role in supporting biodiversity and rural livelihoods, to explore other means for protection of GDEs in politically unstable regions. Our GDE map provides critical information for prioritizing and developing policies and protection mechanisms across various local, regional or international scales to safeguard these important ecosystems and the societies dependent on them.

## Abstract (web summary)

Mapping of groundwater-dependent ecosystems, which support biodiversity and rural livelihoods, shows they occur on more than one-third of global drylands analysed, but lack protections to safeguard these critical ecosystems and the societies dependent upon them from groundwater depletion.

## Main

Globally, groundwater is critical for meeting human and ecosystem water needs, especially in drylands, which comprise roughly 40% of global land area and support more than two billion people. Serving as a buffer when surface water and precipitation are insufficient, groundwater is particularly relied on in dryland regions and increasingly important in meeting higher water demands under a warming climate^5–7^. Despite groundwater accounting for most liquid freshwater on Earth, groundwater depletion is occurring rapidly in many places throughout the globe^8–10^. When groundwater depletion occurs, groundwater levels can drop out of reach from wells^11^ and ecosystems^12–14^, creating a lack of access to drinking or irrigation water and causing or contributing to land subsidence, seawater intrusion, streamflow depletion, ecosystem decline and biodiversity loss^12–15^. Ecosystems are particularly susceptible to groundwater depletion because legal protections and environmental water rights are lacking around the globe^1,16^, and environmental groundwater requirements are often overlooked by conservationists, water managers and human development organizations^2,17^.

Ecosystems relying on groundwater for some or all of their water needs are collectively referred to as groundwater-dependent ecosystems (GDEs). Although GDEs occur across many biomes, they are of greatest concern in drylands, where near-surface water availability is limited compared to humid environments. Water availability within dryland GDEs varies through time and space, as water tables rise and fall, generating surface flow in intermittent and perennial streams, while also providing water to unsaturated soils occupied by the roots of numerous plant species. Under natural conditions, the water table level fluctuates in response to seasonal and interannual climate forcings, resulting in spatially and temporally dynamic interconnections with plant roots and surface water. Natural variations in water availability support highly diverse ecosystems in which groundwater provides a reliable water supply, thermoregulation and/or unique habitat conditions depending on the expression of groundwater on, near or within the Earth’s surface^18^. In drylands, GDEs are important ‘island’ ecosystems that are often isolated by the surrounding xerophyte-dominated desert environment^19^. GDEs are often biodiversity hotspots with niche habitats that support rare and endemic species, and provide critical thermal and hydrologic refugia during dry seasons, droughts and long-term climate changes^20^. However, perturbations in groundwater quantity and quality regimes due to climate change and other anthropogenic stressors such as pumping are placing these GDE biodiversity hotspots under threat, which can result in a cascading series of negative effects on GDEs ranging from short-term water stress to the permanent loss of species and habitats. In addition, effects on GDEs can adversely affect a wide range of benefits they provide to society, including subsistence livelihoods, water quality regulation, streambank stabilization, flood risk reduction, climate regulation, recreational opportunities and cultural values^21,22^.

Knowing the location and extent of GDEs is a critical first step to monitor, manage and protect these important ecosystems. Nevertheless, spatial data on GDEs are lacking in many places globally. GDE mapping so far has been predominantly a localized process requiring time-consuming data collection, expert review and field studies to verify ecosystem access to groundwater. At the same time, GDE mapping at broader landscape scales (more than 50 km^2^) has become increasingly possible through remote sensing and spatial analyses^3,23^. GDE mapping on broad scales has been conducted in Australia^3,24^, California^25^, Central Asia^26^, Chile^27^, Oregon^28,29^, Nevada^30^, Netherlands^31^, Ireland^32^, South Africa^33^, Spain^34^ and Texas^35^. The most common GDE mapping methods use inference-based approaches, which rely on landscape indicators that include hydrologic features (for example, springs, wetlands and rivers supported by baseflow), and vegetation mapping from aerial or satellite imagery^23^. Recent advances in remote-sensing techniques, cloud computing, emerging datasets and machine learning have markedly improved land cover, vegetation and climate mapping over large spatial scales. However, machine learning applications for mapping GDEs have remained limited to specific geographic locations^24,26,27,34^, or at a coarse resolution (roughly 1 km) globally^2^. It is imperative that the global distribution and extent of GDEs be improved so that programmatic and policy decisions can protect these vulnerable dryland environments at appropriate management scales.

Here, we use a random forest machine learning model to provide a high-resolution (1 arcsecond, roughly 30 m at the equator) spatially explicit global map of probable GDEs in dryland regions. The goals of our map are to: (1) generate a conservative (low) estimate of the likely presence and extent of GDEs; (2) provide a reproducible methodology that allows for periodic mapping to detect changes over time, and which can be refined for regional GDE mapping efforts at various scales using local data and expertise, as well as high-resolution satellite imagery; and (3) serve as a starting point for prioritizing policy and programmatic decisions to enhance GDE monitoring and in situ validation studies so that GDEs can be protected by relevant groups, organizations and governments across the globe. Our results show that more than half (53%) of mapped GDEs are potentially threatened by groundwater depletion and only 21% of GDEs exist on protected lands or in jurisdictions with sustainable groundwater management policies. Because GDE protection may need to be achieved through integrated policies or programmatic work instead of sustainable groundwater management laws that may not be tractable in politically unstable regions, we also examined the linkage of mapped GDEs with cultural and socio-economic factors within the Greater Sahel region of Africa. Finally, we discuss how our global GDE map and methodology can be used as a starting point to facilitate and improve policy and programmatic decisions at the local level.

## High-resolution GDE mapping

**Extended Data Fig. 1:**
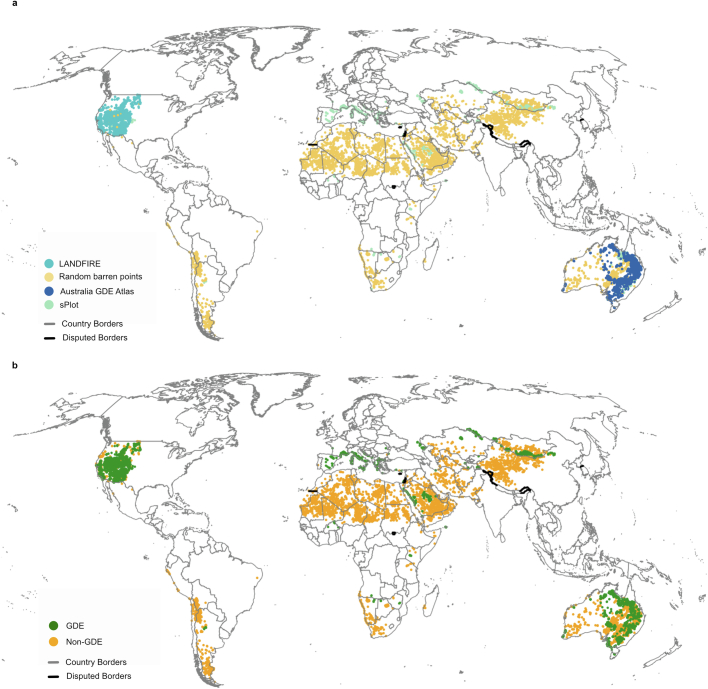
Groundwater-dependent ecosystem (GDE) training and validation data (*n* = 34,454 points). (a) Data sources: LANDFIRE vegetation (*n* = 6,652 points), Australian GDE Atlas data (*n* = 19,111 points), ESRI 10 m land use land cover bare ground data (*n* = 4,075 points), and sPLOT vegetation (*n* = 4,616 points). (b) GDE (*n* = 16,805 points) and non-GDE (*n* = 17,649 points) classifications.

We combine 6 years (2015–2020) of Landsat 8 imagery, climate, topographic, groundwater and GDE training data (*n* = 34,454 training points; Extended Data Fig. 1 and Extended Data Table 1) to map the likely presence of both aquatic and terrestrial GDEs at roughly 30 m resolution across global drylands. Within our random forest model, training data are used within an ensemble of decision trees to perform a supervised classification resulting in each pixel being classified as a GDE or non-GDE. Given the global scale of our study and reliance on satellite-based indicators, this binary classification (GDE or non-GDE) occurs regardless of whether the GDE is aquatic or terrestrial, and slightly dependent (facultative) or entirely dependent (obligate) on groundwater but excludes subterranean GDEs that exist within aquifer formations. Characterizing the timing and nature of groundwater dependence requires intensive in situ field monitoring, such as isotopic studies that require localized field sampling and are not feasible at the global scale. Thus, the intention of our map is to provide an indication of where GDEs are most likely to exist across global drylands, and to provide a starting point for regionally refined mapping efforts and verification using local datasets, knowledge and targeted follow-on work.

**Extended Data Fig. 2:**
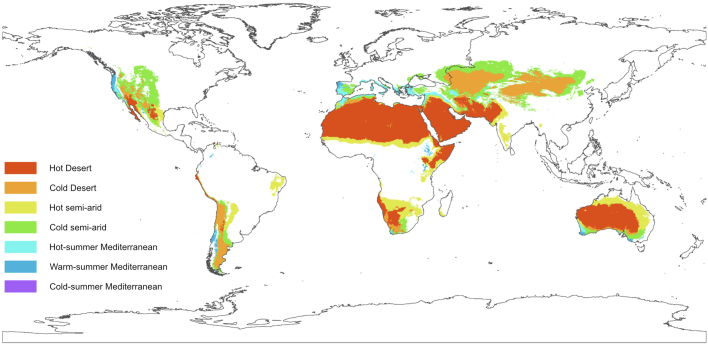
Köppen-Geiger climate classifications used to designate dryland regions for GDE mapping. Data Source: Beck et al., 2018.

**Extended Data Fig. 3:**
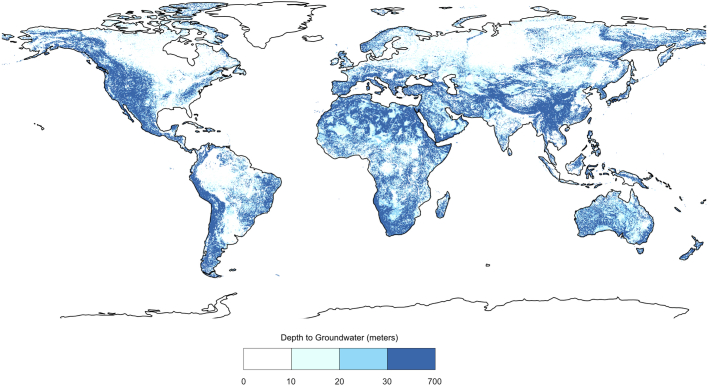
Global depth to groundwater (≥30 meters). Masked areas where depth to groundwater >30 meters is denoted in dark blue and were not included in the model. Data Source: Fan et al. 2017.

In the absence of a comprehensive global groundwater level database, our random forest model uses publicly and globally available satellite-based data, including vegetation and water indices, ambient land surface temperature (LST), climate and topographic data (Methods). To infer whether ecosystems are being supported by groundwater, our approach assumes that ecosystems with access to groundwater will appear as ‘blue or green islands’ because they will be wet and maintain ecohydrologic and photosynthetic function during the dry season, in contrast to those without access to groundwater^23^. For this reason, we selected satellite-based data that can measure vegetation greenness, leaf water content, open water bodies, the ratio of the annual sum of actual plant transpiration to precipitation (ETaP) and the spatial anomaly of LST. ETaP distinguishes pixels in which plant transpiration exceeds precipitation, indicating a likely reliance on groundwater, and LST distinguishes GDEs based on their cooler temperatures relative to the surrounding environment. These cooler temperatures are driven by higher evaporative rates from soil and water bodies influenced by groundwater and higher transpiration rates due to a more abundant water supply available to phreatophytic vegetation^36^. Although GDEs exist in both wet and dry environments, the identification of GDEs in humid environments is more difficult using existing satellite-based data because of the inability to differentiate between precipitation and groundwater sources. Thus, we restrict this inference-based approach and the model extent to global drylands (Extended Data Fig. 2), and exclude places with deep groundwater that are outside the reach of most plant roots^37^ (more than 30 m, Extended Data Fig. 3), in addition to agricultural and urban lands. This resulted in a total model analysis area of 23.2 million km^2^. Because our model relies on satellite-based thermal and spectral data from the 2015–2020 period, the resultant map reflects the likely location of aquatic and terrestrial GDEs for this snapshot in time.

**Fig. 1:**
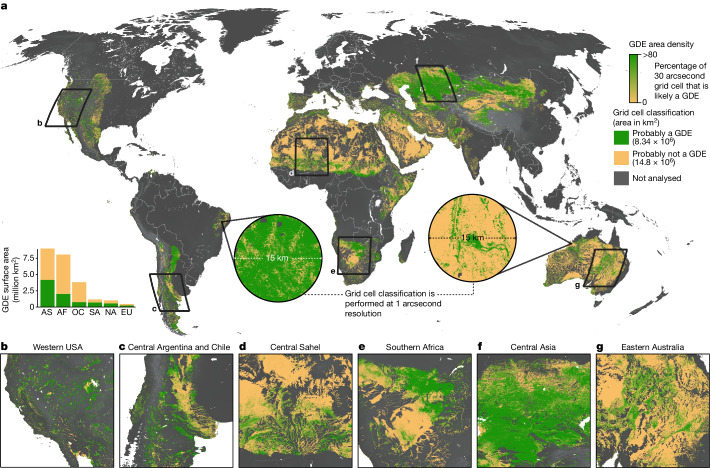
Global GDE map. **a**, Global map shows GDE area density at 30 arcsecond resolution (roughly 1 km grids). Call-out circles show binary GDE classification at the full 1 arcsecond resolution (roughly 30 m grids). Bar plot (bottom left) shows GDE surface area distribution across continents. AS, Asia; AF, Africa; OC, Oceania; SA, South America; NA, North America; EU, Europe. **b**–**g**, Regional maps shown at the full 1 arc second resolution for the western USA (**b**), central Argentina and Chile (**c**), the central Sahel region (**d**), southern Africa (**e**), central Asia (**f**) and eastern Australia (**g**). The global map is shown in the Robinson projection whereas all panel insets are shown in geographic projection (latitude and longitude) referenced to the World Geodetic System (WGS) 1984 datum. An interactive version of the full resolution map is available at https://codefornature.projects.earthengine.app/view/global-gde.

**Extended Data Fig. 4:**
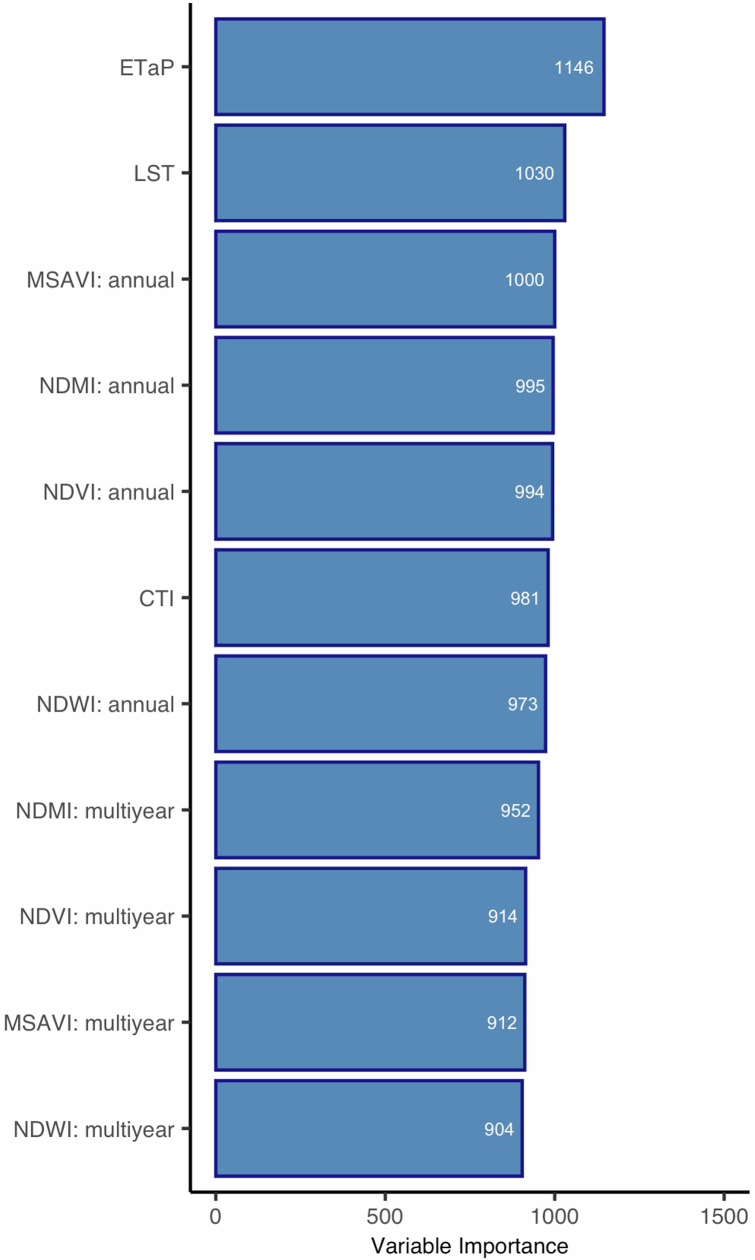
Random forest variable importance plot. Random forest variable importance plot ranked highest to lowest: ratio of the annual sum of plant transpiration and precipitation (ETaP), ambient land surface temperature spatial anomaly (LST), Landsat modified soil adjusted vegetation index annual average (MSAVI: annual), normalized difference moisture index annual average (NDMI: annual), normalized difference vegetation index annual average (NDVI: annual), compound topographic index (CTI), normalized difference water index annual average (NDWI: annual), normalized difference moisture index inter-annual variability (NDMI: multiyear), normalized difference vegetation index inter-annual variability (NDVI: multiyear), Landsat modified soil adjusted vegetation index inter-annual variability (MSAVI: multiyear), and normalized difference water index inter-annual variability (NDWI: multiyear).

**Extended Data Fig. 5:**
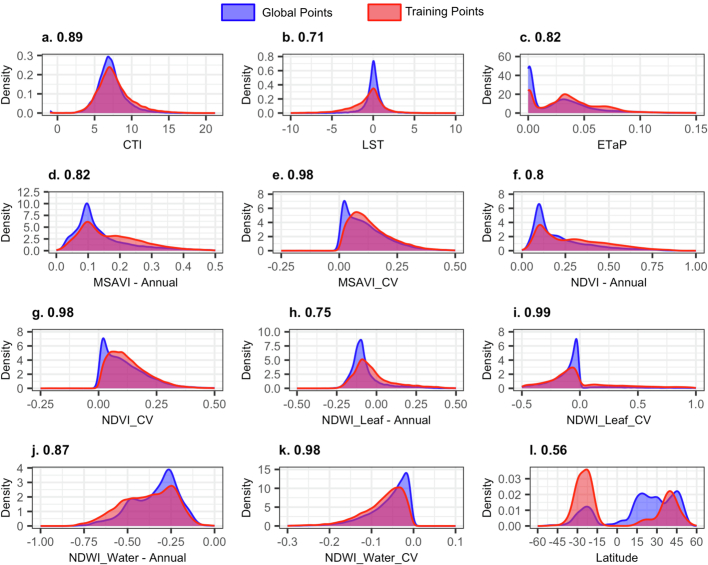
Predictor variable distribution plots comparing predictor values for the main model’s training and validation data (*n* = 34,454 points) and random global points within the model extent (*n* = 32,954 points). The numbers in the upper left of each plot indicate the degree of overlap between the global and training point distributions, with zero indicating no overlap and one indicating complete overlap.

**Extended Data Fig. 6:**
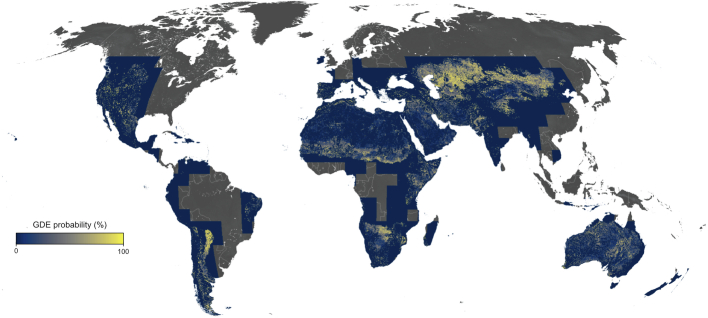
Groundwater-dependent ecosystem (GDE) probability map, indicating how likely a pixel is a GDE (100%) or non-GDE (0%) within the model extent at 1 arcsecond (~30 m) resolution. Probability data are provided in 5 degree x 5 degree tiles (see Data availability). Areas outside of these tiles extent are shaded in gray. The GDE probability data map is also available as an interactive web map (https://codefornature.projects.earthengine.app/view/global-gde).

The validation accuracy of the random forest model was 84%, which is a measure of how well the model predicted true positives (GDEs) and true negatives (non-GDEs, Extended Data Table 2). The model precision, which measures the percentage of the predicted GDEs that are actually GDEs (true positives), was 81%. The model recall, which measures the percentage of actual GDEs that were predicted correctly, was 87% (Extended Data Table 2). The two most important predictor variables for distinguishing GDEs from their surrounding environment were ETaP and LST (Extended Data Fig. 4). To evaluate how well the model performs within regions lacking training data within the model extent (Extended Data Fig. 1), we compared the distribution of the predictor variables results from our model training data points (*n* = 34,454 points) with a randomly generated global point dataset of comparable size (*n* = 32,954 points). The distributions of each of the 11 predictor variables were similar across the training and global points, with their overlap index (Methods) ranging between 71% and 99% (Extended Data Fig. 5). Furthermore, regional cross-validation tests, which are a standard machine learning protocol for assessing model performance in areas without training data, were performed in the Sahel, Western Australia and New Mexico (USA) yielding validation accuracies of 69, 53 and 61%, respectively (Supplementary Tables 1–3). Precision was much higher than recall in the Sahel and Western Australia cross-validation tests, but lower in New Mexico. The lower recall rates in our cross-validation tests are a result of GDE training points being misclassified as non-GDEs, which suggests that our model is probably under-classifying GDEs and thus provides a conservative (low) estimate of the likely presence of GDEs within dryland regions worldwide. One possible explanation is that GDEs in drylands can be sparsely vegetated or contain small springs that may be difficult to detect at 30 m resolution. For example, if ground-truthed training points used in our model contain a lone tree or small spring within a roughly 30 metre pixel, the pixel can be saturated by bare ground reflectance that would result in that GDE point being misclassified as a non-GDE grid cell. Thus, it is possible that grid cells classified as non-GDEs may in fact be a GDE, especially in more arid landscapes in which GDE features are likely to be smaller and more difficult to detect with remote-sensing data. To better characterize the uncertainty of our model, we also generated a probability layer in our GDE map that contains the likelihood that each pixel is a GDE (100%) or non-GDE (0%) (Extended Data Fig. 6 and Methods). In our GDE map (Fig. 1), we differentiated likely from non-likely GDE grid cells using a likelihood threshold of 50% but end-users of our data can reduce this threshold to lower values if less-conservative estimates of GDE presence are desired.

Our mapping reveals that GDEs are probably present within 8.34 million km^2^ of global drylands, comprising 36% of the global dryland area analysed here (Fig. 1). An interactive version of the high-resolution (1 arcsecond, roughly 30 m) spatially explicit global GDE map and probability layer are accessible as a web map (https://codefornature.projects.earthengine.app/view/global-gde). GDEs coincide with many global biodiversity hotspots, such as the California Floristic Province, Mesoamerica, Tropical Andes, Central Chile, Mediterranean Basin, Eastern Arc and Coastal Forests of Tanzania/Kenya, Caucasus, Indo-Burma, Southwest Australia and New Zealand^4^. Mapped GDEs include a wide range of terrestrial and aquatic ecosystem types, including phreatophytic vegetation, rivers and streams, springs and wetlands that not only support rare and endemic species, but also rural livelihoods that depend on GDEs for domestic water supplies, food and livestock forage (Supplementary Fig. 1).

## Groundwater development linkages

**Fig. 2:**
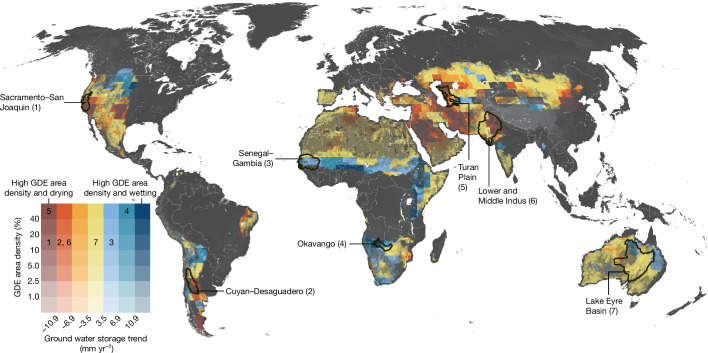
GDE area density and regional groundwater storage trends. Global relationship between GDE area density and groundwater storage trends at 30 arcminute resolution (roughly 50 km grids). Annotated numbers inside the legend correspond to the area-weighted average values per freshwater ecoregion (Methods) highlighted in the map.

**Extended Data Fig. 7:**
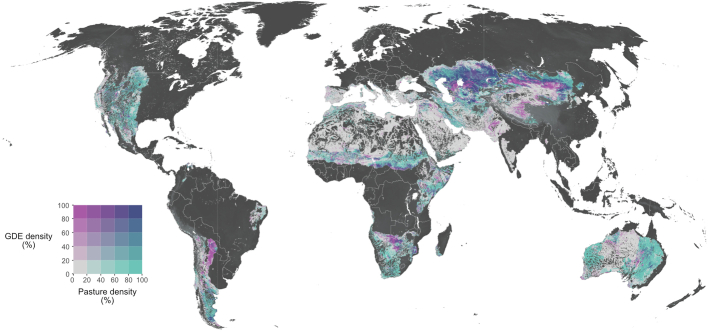
Groundwater-dependent ecosystems (GDE) area density intersected with pastoral lands area density. Globally, our map indicates 59% of GDEs (4.9 million km^2^) overlap lands with >25% pastoral land density at 5 arcminute resolution.

To assess risks to GDEs posed by groundwater depletion, we compared GRACE-derived groundwater storage trends over the past 20 years (2002–2022) for mapped GDEs, which reveal important differences between continents. For example, mapped GDEs are more contiguous and are more extensive in Central Asia, the Sahel and South America (Fig. 1), where they coincide with pastoral landscapes (Extended Data Fig. 7) and lower rates of groundwater depletion (Fig. 2). This is in contrast to more fragmented GDE landscapes in Australia and North America where agricultural lands and groundwater pumping dominate^38^. Globally, our map indicates 59% of GDEs overlap lands with more than 25% pastoral land use (among areas with pastoral land use data). Because many GDEs rely on shallow groundwater, regions with a history of groundwater pumping are likely to have lost many GDEs over the decades since pumping commenced^12,13,39^. For example, intensive groundwater pumping in California’s Central Valley has caused groundwater levels to drop below the roots of plants and to become disconnected from stream channels, contributing to a landscape with highly fragmented GDEs that often rely on shallow groundwater supported by local irrigation return flow, water conveyance or discharge from wastewater treatment facilities^15^. As groundwater depletion continues to increase globally to meet human^40^ and atmospheric evaporative^41^ demands from a warming climate, less groundwater will be available for GDEs to cope and buffer against reduced surface water availability and increased plant water stress^6^.

Globally, more than half (53%) of mapped GDEs (3.81 million km^2^) exist within regions showing declining groundwater storage trends (among GDE areas with available data; 7.20 of 8.34 million km^2^; Methods). However, there is significant regional variability in the threats posed to GDEs by groundwater depletion. Regions where most (more than 50%) dryland GDEs are in areas experiencing groundwater storage loss include Europe (90%), Asia (75%) and North America (65%). Conversely, only moderate and small percentages of GDEs in South America (37%), Oceania (29%) and Africa (17%) are facing similar threats (Fig. 2). Because global groundwater storage trend data are only available at coarse spatial resolutions and vertically integrate shallow and deep groundwater resources (Methods), the direct impact on GDEs will vary considerably at local scales not captured in the large-scale storage trend data. Groundwater storage loss can result in deeper water tables and reduced groundwater flow across the landscape and at the intersection of surface water bodies, but will vary locally depending on the hydrologic regime, aquifer configuration and streambed hydraulic conductivity. However, the widespread occurrence of groundwater storage losses in regions with identified GDEs underscores the need to proactively protect these ecosystems from the threat of groundwater depletion in regions not facing the same storage losses, such as found across much of Africa. In many regions around the world, GDEs lack protection and pressures on GDEs are exacerbated by complex cultural, socio-economic and political factors.

## Cultural and socio-economic linkages

To illustrate the linkages between GDEs with cultural and socio-economic factors, we focus on the Greater Sahel region in which GDEs play an essential role in supporting biodiversity, rural livelihoods and providing sustenance and relief along human migration pathways for pastoralists and traders^42^. With half of the world’s poor living in sub-Saharan Africa, the Sahel is a fragile region laden with social and climate instability, including social conflict, food insecurity, human displacement and extreme flood and drought events^43^. In the aftermath of severe drought events during the 1970s and 1980s, competition over water and agricultural resources between nomadic herders and sedentary farmers spurred ongoing confrontations for water, crop land and grazing options across the region^44^. During dry periods, when herds can no longer rely on nutrient-rich annual grasses, pastoralists move their herds onto croplands to graze and browse within wetlands and on trees, shrubs and perennial grasses that are probably sustained by groundwater, which can exacerbate conflict^45,46^.

**Fig. 3:**
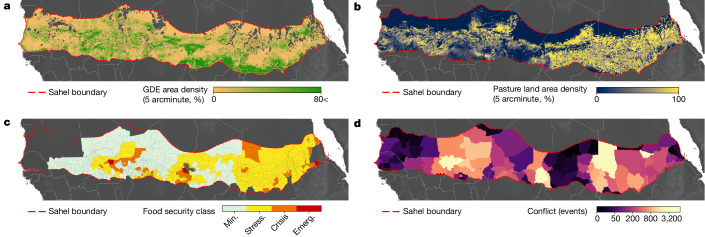
The fragility of GDEs and associated communities within the Greater Sahel. **a**, GDE area density at 5 arcminute resolution. **b**, Pastoral land area density at 5 arcminute resolution. **c**, District-level food insecurity classes as of October 2021. Food insecurity classes are Min., Minimal; Stress., Stressed; Crisis; Emerg., emergency. **d**, Armed conflict location and event data (all events between January 1997 and February 2021) summarized for GADM level 1 administrative areas. Data sources from **b**–**d** are provided in Supplementary Table 6.

In the Greater Sahel, our findings show that four well-known conflict hotspots (the Liptako–Gourma region at the borders of Mali, Burkina Faso and Niger; the Lake Chad Basin at the borders of Chad, South Niger, Northern Nigeria and Cameroon; the Darfur region at the borders of Sudan, South Sudan, Chad and the Central African Republic; and the South Kordofan region between Sudan and South Sudan) have a high prevalence of GDEs, which support local livelihoods and exist at the convergence of forced migration pathways^47^. These hotspots coincide with growing food insecurity in the wake of climate shocks and conflict that have resulted in the expansion of crop cultivation into traditional grazing areas^48^ (Fig. 3). The overlap between GDEs and conflict zones of social vulnerability emphasizes the importance of recognizing the interdependencies between GDEs, climate change, rural livelihoods, food security and social stability in subnational, national and regional protection strategies. This is particularly important because many of our globally mapped GDEs co-exist with pastoral lands (Extended Data Fig. 7), where GDEs are likely to provide critical ecosystem services for both wildlife and livestock. However, our results also indicate that these same GDEs, and the services they provide, are likely to be threatened by policies that encourage groundwater exploitation due to agricultural intensification. For example, single-objective policies aimed at food security that promote the proliferation of groundwater wells for irrigation or food pricing that encourages water-intensive grain cultivation have exacerbated groundwater depletion in regions such as India^49^. The likelihood of similar unintended consequences of single-issue policies is high for regions such as the Sahel, and groundwater depletion that leads to GDE degradation stemming from well-meaning policies (for example, borehole development for irrigation) could contribute to further regional destabilization by excluding pastoralists and increasing their societal vulnerability to climate shocks. Thus, multi-disciplinary approaches are necessary to address the interdependence of economic development, natural resources and conservation, to ensure that diverse livelihoods and communities surrounding GDEs in dryland areas are protected along with these critical natural environments.

## Overcoming global conservation challenges

GDEs in drylands are at risk of severe ecologic damage and loss if policies, development projects and management actions do not explicitly factor in environmental groundwater needs^14,17^. In the race to combat climate change and unprecedented biodiversity loss, global initiatives and land protection often overlook the significance of groundwater in supporting important species, habitats and many critical functions including climate regulation^2,22^. The importance of groundwater is generally under-represented in the United Nations Sustainable Development Goals, with vague linkages to ecosystems under Target 6.4 (Water use and scarcity) and Target 6.6 (Water-related ecosystems). Although environmental water needs for GDEs are increasingly being protected under Australia’s Environment Protection and Biodiversity Conservation Act, and considered under sustainable water policies, such as Australia’s National Water Initiative, the European Union’s Water Framework Directive and California’s Sustainable Groundwater Management Act, significant policy gaps remain globally.

**Fig. 4:**
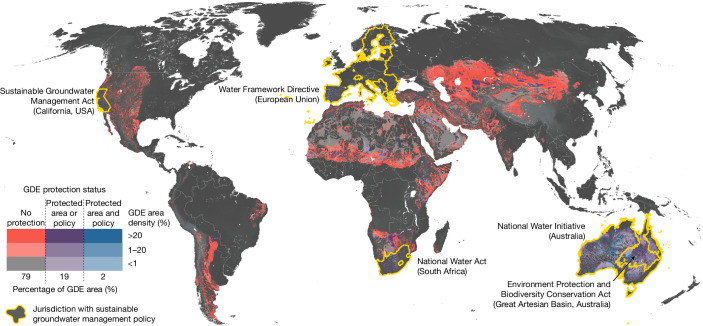
Protection status of GDEs. The proportion of mapped GDEs with no protection (red) is 79%, with the remaining 21% having some degree of protection (blue and purple). GDEs shown in purple exist on protected areas or in jurisdictions with sustainable groundwater management policies. GDEs shown in blue are protected by both measures (protected area and sustainable groundwater management policy). GDE area density is shown in this figure at 30 arcsecond resolution (roughly 1 km grids).

Our results show that only 21% of globally mapped dryland GDEs (1.76 million km^2^) have some degree of protection (Fig. 4). However, even in places with well-established legal frameworks that limit groundwater development, the implementation of these policies often falls short of protecting ecosystem water needs^50^. For example, a common practice within groundwater law is to manage groundwater towards a safe yield, which considers groundwater usage to be safe if it falls within the natural recharge rate^51^. However, the concept of safe yield fails to acknowledge negative ecologic consequences^17^. Even jurisdictions that have adopted a more inclusive definition of sustainability, such as in Australia, California in the USA and the European Union where ecologic water requirements or an evaluation of ecosystem effects are required, are falling short of meeting ecosystem water needs. This is due to inequitable decision-making processes that prioritize human over ecosystem water needs, the absence of environmental groundwater rights regimes, limited ecohydrologic expertise in water agencies and a lack of scientific consensus on what measurable groundwater targets and thresholds are representative of environmental water needs^1,14,16,17,50^. Even with improvements, groundwater laws that limit groundwater development or call for sustainable groundwater management planning may be intractable in politically unstable regions, as illustrated for the Sahel. Thus, it may be necessary to achieve GDE protection through means of other local, regional or international policies or humanitarian efforts.

Our study provides a conservative map of GDEs in drylands globally and an approach to delineate GDEs at local scales. However, further ground-truthing and verification should be undertaken before applying the global map to local contexts. Our map nevertheless provides critical information for subnational, national and intergovernmental organizations to prioritize, conceptualize and develop policy and protection mechanisms, so that efforts can be made to safeguard and avoid further degradation to these important dryland ecosystems and the communities that depend on them.

## Methods

### Model development

Data processing and modelling were conducted in Google Earth Engine (GEE), an application program interface that provides access to large publicly available datasets and machine learning algorithms, which enables complex computing across large spatial and temporal scales that was nearly impossible in the recent past^52^.

#### Model extent

Dryland regions were identified at 30 arcsecond (roughly 1 km) resolution using the Köppen–Geiger climate classes: arid and semi-arid (Type B), and three temperate climate types with distinct dry summer seasons (type C)^53^ (48.5 million km^2^, Extended Data Fig. 2). Agricultural and urban areas were masked out using the Environmental Systems Research Institute (ESRI) roughly 10 m resolution global land use and land cover map, which were derived from deep learning models and 2017–2020 Sentinel-2 imagery^54^. Isolated patches of groundwater-dependent vegetation existing within agricultural lands may be classified as croplands and subsequently masked out of the model extent. Oceans and inland seas were masked out using the Copernicus Global Land Service Dynamic Land Cover map at 100 m resolution (CGLS-LC100), which is based on 2015–2019 Sentinel imagery^55^. Global depth-to-groundwater (DTG) data at roughly 1 km spatial resolution^56^, were used to define the model extent by masking out pixels where DTG exceeded 30 m from the land surface (Extended Data Fig. 3), which is beyond the rooting zone of most phreatophytic vegetation^37^. As the DTG dataset contains data gaps where open water occurs, we assigned a DTG value of 0 for pixels identified in the ESRI dataset as open water. Next, the DTG layer was smoothed using a 1.5 pixel focal mean window to interpolate values for any remaining, isolated ‘no data’ pixels using surrounding pixel values. The small window size was used to minimize the effects of smoothing on DTG values in regions with large changes in surface elevation. On visual inspection, the remaining ‘no data’ gaps appear to surround water sources that had been identified using the land cover data. It was assumed that DTG is shallow in these areas, and these remaining pixels were also assigned DTG values of zero. Only pixels with DTG less than or equal to 30 metres were included in the analysis to map GDEs. After applying these various spatial masks, the total model extent is 23.2 million km^2^.

#### Training and validation data

Training and validation GDE data (Extended Data Fig. 1 and Extended Data Table 1) were derived from ground-truthed points within the public version of the LANDFIRE 2016 Remap Reference Database (LFRDB)^57^, the Australian Groundwater Dependent Ecosystem Atlas^58^ and the sPLOTOpen dataset^59^. In the Australian GDE Atlas, subterranean GDEs from karsts were excluded and the remaining aquatic and terrestrial GDE data were considered as GDE if classified as a ‘Known GDE—from regional studies’, and as non-GDE if classified as a ‘Low potential GDE—from regional studies’. Ground-truthed vegetation data inventoried within the LFRDB and sPLOTOpen datasets were classified as GDE or non-GDE data according to species and location based on expert and literature review (Supplementary Table 4). For the LFRDB dataset, phreatophytes were classified from four different states in the western United States: Arizona, California, Nevada and Oregon. If there was consensus among two or more states that a particular plant species in the reference database was a phreatophyte, then it was classified as a GDE. Non-GDE points were identified when a plant species was not identified as a phreatophyte in three or more states. Other non-GDE training points were created by randomly sampling barren areas (*n* = 10,000 points) within the ESRI 10 m land use and land cover map. Because our model relies on satellite-based thermal and spectral data, we intentionally selected training data, predictor variables and regions that could readily map ecosystems showing surface expressions of groundwater. And thus, our GDE map does not reflect GDEs in subterranean systems or in cold or humid environments.

#### Predictor variables

GDEs were mapped globally using 11 predictor variables from a combination of observational, model-based and remote-sensing data, as summarized below.

First, satellite-based indices were developed using roughly 30 m surface reflectance data from Collection 2 of the Landsat 8 satellite platform. All satellite images were processed in GEE. Landsat 8 data in GEE contains atmospheric-corrected multispectral imagery^60^, and contains a quality assessment band with cloud mask information (‘QA_PIXEL’) that is available for users to identify cloudy and cloud-free pixels. Landsat scenes with greater than 20% cloud cover were not included in the analysis to minimize misclassification of GDEs. For scenes with less than or equal to 20% cloud cover, clouds, snow and/or ice and cloud shadows were masked using the CFmask algorithm^61–64^. Four satellite-based vegetation and water indices were calculated: (1) normalized difference vegetation index^65^, a measure of greenness; (2) normalized difference moisture index^66^, a measure of water in plant mesophyll, (3) normalized difference water index^67^, a measure of open water and (4) modified soil adjusted vegetation index^68^, a measure of greenness that minimizes soil brightness effects on the vegetation signal (Supplementary Table 5). For each of these indices, two metrics were developed to be used as predictors in the random forest model using multi-year (2015–2020) satellite imagery from the dry season (late summer and early autumn period). Dry season satellite images were selected because GDEs can be more readily distinguished from non-GDEs as GDEs’ reliance on groundwater allows them to maintain vegetation vigour later into the season, when surface water and precipitation are scarce^15,69^. The dry season period was defined as 1 July–30 September in the Northern Hemisphere, and 1 January–31 March in the Southern Hemisphere. The two metrics developed for each index were (1) annual dry season average, and (2) multi-year coefficient of variation of the average dry season period as a measure of interannual variability. The four indices with two metrics each resulted in eight predictor variables. The coefficient of variation, which is calculated as the ratio of the standard deviation to the mean, was chosen over the standard deviation to provide a fairer measure of variability, given that pixels with high vegetation cover will have a higher variation than pixels with lower vegetation cover.

Second, the ratios of annual sums of ETaP, averaged over the 2003–2016 time period for which vegetation transpiration data were available, were included as a predictor variable to indicate groundwater dependence in which annual vegetation consumptive water use exceeded precipitation. This exceedance (that is, ETaP greater than 1) indicates that plant water needs are probably being met by groundwater rather than infiltrated precipitation. Transpiration to precipitation ratios were calculated in GEE using 500 m resolution vegetation transpiration data from the Penman-Monteith-Leuning Evapotranspiration V2 (PML_V2) product^70,71^, and 1/24° resolution precipitation data from TerraClimate^72^.

Third, compound topographic index (also known as topographic wetness index) data distinguish between ridge and valley forms, and were used to indicate the likelihood that soil is saturated with water as a result of topographic position without accounting for climate factors^73^.

Fourth, an ambient LST spatial anomaly dataset was developed using the Landsat dataset described in point (1) to identify anomalously cool or warm places relative to their surroundings, which is an expected attribute of GDEs. The surface temperature quality assessment band (‘ST_QA’), which indicates uncertainty about temperatures given in the surface temperature band file, was used to eliminate pixels with uncertainties greater than 5 °C. The spatial anomaly dataset was derived by calculating the differences in LST between a given focal pixel and the average LST of all pixels within the surrounding 270, 2,700 and 5,400 m^2^ area. The three differences were then averaged to generate a multi-scaled result^74^. From there, the 5 year average (2015–2020) of the annual mean summer and/or early autumn (fall) period LST spatial anomaly was calculated. Before applying the algorithm, open water land cover types were masked out to eliminate their influence on the spatial anomaly calculations.

Distribution plots for each of the 11 predictor variables were created to compare the training data (*n* = 34,454 points) with randomly generated global points within the model extent (*n* = 32,954 points). Overlap statistics were calculated in the R statistical software using the overlapping package^75^, in which a statistical value of zero represents no overlap between the two samples’ distributions and a statistic value of one represents complete overlap (that is, identical datasets).

#### Random forest algorithm

**Extended Data Fig. 8:**
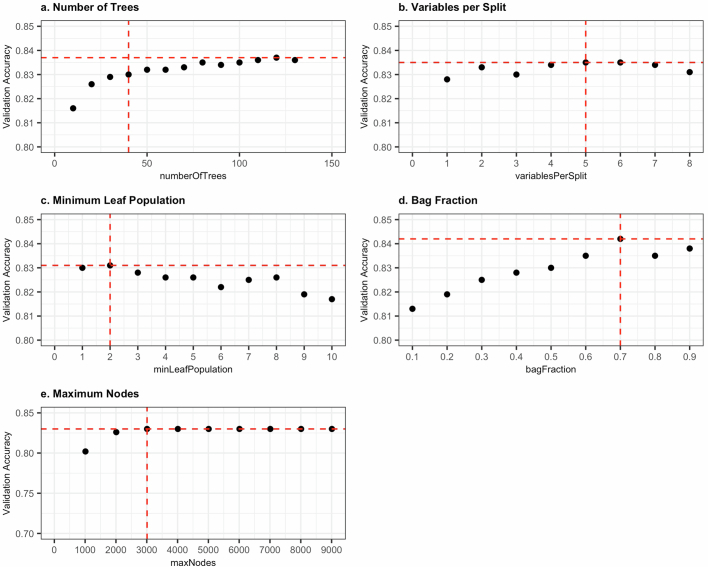
Hyperparameter Tuning for the main Global GDE model. Selected hyperparameter values are indicated by the red vertical dashed lines. The red horizontal dashed lines represent the highest accuracy of parameter values.

We determined the likely presence of GDEs globally using a random forest algorithm within GEE based on the predictor variables, and training and validation data introduced above. The random forest algorithm is a statistical model that trains an ensemble of classification and regression tree models populated by random subsets of the model calibration data and predictor variables^76^. The trees within random forest are created through a ‘bagging approach’ that draws a random subset of attribute data (that is, a selection of predictor variables) through replacement, resulting in some samples to be selected several times and others never selected (the out-of-bag fraction). The ‘bagging approach’ and attribute sampling both help ensure that each decision tree is independent of each other, which helps to minimize overfitting in the random forest model when the majority decision is taken from the ensemble of trees^77^. Random forest modelling was selected because it is computationally efficient, less likely to overfit and can handle many predictors^78,79^. The model was trained on 34,454 point locations of aquatic features and vegetation types known to rely on groundwater (Extended Data Table 1 and Extended Data Fig. 1). The data were split 80 to 20 for training and test sets. Hyperparameter tuning was used (Extended Data Fig. 8), resulting in the model to contain 40 trees (numberOfTrees), five variables per split (variablesPerSplit), two minimum leaf population (minLeafPopulation), 0.7 bag fraction (bagFraction) and 3,010 maximum number of nodes (maxNodes). The out-of-bag error estimate was 0.18. Outputs from the random forest model include a ‘soft’ probability class (Extended Data Fig. 6) varying between 0 and 100% using a probabilistic mode in the random forest model (setOutputMode ‘MULTIPROBABILITY’), and a ‘hard’ probability class that results in a binary GDE (1) and non-GDE (2) classification that is obtained by identifying the most accurate soft probability GDE classification using a dynamic thresholding analysis (Fig. 1).

Regional cross-validation tests were performed to further evaluate how well the model extrapolated into regions without training data. This was accomplished by running the model three more times using (1) new training data provided by the World Bank from the Sahel region in Africa^47^ and (2) by omitting our training data from Western Australia and New Mexico, USA to test model performance in these regions. Hyperparameter tuning was used separately using the Western Australia and New Mexico cross-validation training, as those cross-validation tests used a subset of the main model training and validation data, whereas the Sahel cross-validation test used the main model’s training and validation data. It is important to note, that the GDE data from the Sahel are not ground-truthed data and primarily derived from a literature review, which required us to randomly generate points within polygon features and line buffers, which very probably introduced some uncertainty into this dataset. For this reason, the Sahel data were not incorporated into the random forest classifier, and only used as a validation outside the model. Hyperparameter tuning for the Western Australia cross-validation test (Supplementary Fig. 2), resulted in the model to contain 70 trees (numberOfTrees), two variables per split (variablesPerSplit), one minimum leaf population (minLeafPopulation), 0.9 bag fraction (bagFraction) and 3,010 maximum number of nodes (maxNodes). Hyperparameter tuning for the New Mexico cross-validation test (Supplementary Fig. 3), resulted in the model to contain 40 trees (numberOfTrees), six variables per split (variablesPerSplit), one minimum leaf population (minLeafPopulation), 0.7 bag fraction (bagFraction) and 3,010 maximum number of nodes (maxNodes).

### Post hoc analyses

#### Data summarizing at multiple resolutions

Post hoc analyses were performed at varying resolutions to best match the base resolutions of the datasets the GDE map was compared with. Thus, whereas the core GDE map developed in this study is at 1 arcsecond resolution (roughly 30 m grids at the equator), we also calculated and have provided GDE area densities at 30 arcsecond (roughly 1 km), 5 arcminute (roughly 10 km) and 30 arcminute resolution (roughly 50 km). GDE area densities were derived at each resolution as a ratio of: (1) area analysed per grid cell and (2) total grid cell area. We anticipate that these summary datasets (Data availability section) will be of interest to the broader scientific and practitioner community that routinely operates at these resolutions.

#### Groundwater storage trends

GRACE-based groundwater storage trends were derived using terrestrial water storage anomalies from NASA Jet Propulsion Laboratory Level-3 Release 6 v.2 gridded mascon data (0.5°, roughly 56 km at equator)^80^, and the soil moisture, canopy storage and snow water equivalent time series were obtained from Global Land Data Assimilation System v.2.1 (GLDAS-2.1) Noah^81^ and Variable Infiltration Capacity (VIC)^82^ land surface models. Groundwater storage anomalies are computed by removing the soil moisture, canopy water storage and snow water equivalent anomalies from the terrestrial water storage anomalies based on the modelled water balance^83^, in which the resultant groundwater storage vertically integrates shallow and deep groundwater resources^84^. The groundwater storage trends reported in this study correspond to the April 2002–April 2022 time range.

A limitation of this approach is the lack of representation of surface water anomalies, which are not available at present in an existing global time series data product. However, surface water storage trends are typically small in comparison to large-scale trends in groundwater storage^85^, with a notable exception found in the filling of main reservoirs^86^. The groundwater storage trends provided by this methodology do not cover the entire terrestrial land surface as regions are masked if they contain glaciers whose trends are not accounted for in the above-described methodology. This masking reduces the spatial extent of the groundwater storage trends dataset, with the implication that roughly 1.1 million km^2^ of mapped GDEs exist in these masked out regions. Following our post hoc analysis protocols, our analysis comparing GDE area density with groundwater storage trends is performed at 30 arcminutes to match the resolution of the groundwater storage trend data.

To provide regional summaries of the relationship between GDE area density and groundwater storage trends, we map the relationship between GDE area density and groundwater storage trends globally and calculate area-averaged values for a selection of freshwater ecoregions^87^. We selected freshwater ecoregions as a unit of analysis because they are based on the distribution and composition of freshwater species globally and offer a spatial template that is useful for informing large-scale conservation planning efforts.

### Protected areas

To quantify the extent of GDE protection globally, we compared mapped GDE extents with the World Database on Protected Areas (WDPA)^88^ and to jurisdictions where there are implemented sustainable water policies with GDE protection. The WDPA is the most comprehensive global dataset of protected areas. The WDPA contains both spatially explicit polygon representations of protected area extents as well as points where polygon extents are not available. Although point data correspond to roughly 9% of all entries in the WDPA^89^, we do not account for these areas as doing so requires assumptions on the spatial shape of the protected area. Jurisdictions with sustainable water policies include the European Union, South Africa, Australia and California (USA). We evaluated the protection status of GDEs at 1 arcsecond resolution by rasterizing the WDPA and extents of the aforementioned jurisdictions and compared these extents to the base GDE classification map. We also conducted this comparison at 30 arcsecond (roughly 1 km) resolution for plotting in Fig. 4.

### Limitations

Random forest is an inherently statistical rather than a deterministic, process-based approach that relies on training data and predictor variables to predict outcomes. Like most models, uncertainty can be embedded into models from input variables and training data. By using a random forest model to predict the likely occurrence of GDEs globally, there are three main sources of uncertainty in our final model output: (1) predictor variables: our model uses 11 predictor variables that have complete coverage across the global domain. Each of these predictor variables have different spatial and temporal resolutions (Supplementary Table 6) but each represents the best available datasets for each variable for our global model. Uncertainty embedded in each of the predictor variable datasets can be minimized in local applications of our modelling approach in which higher resolution and local-verified datasets can be used in place of these larger global datasets. (2) Training and validation data: data on the presence and absence of GDEs are limited to specific geographic locations and have temporal resolutions that vary, due to a lack of recognition of GDEs in many jurisdictions (which was a major motivator for this study). The lack of a globally consistent ground-truth dataset and reliance on regional expert opinion to identify GDE versus non-GDE vegetation is another factor that can be improved in more localized applications. (3) Model extrapolation: although we have tuned hyperparameters, checked the distribution of training data with a randomly generated dataset within the model extent for each of our predictor variables and performed regional cross-validation tests, some model extrapolation errors may have occurred. However, our analyses suggest that many of these errors are likely to be underestimating the occurrence of GDEs globally rather than overestimating. This means that whereas there may be pixels designated as non-GDEs, that there may be features (for example, upland channels, forest stands, small springs) within our modelling extent that are groundwater dependent, and vice versa. GDE reliance on groundwater varies in time and space and even for the same species depending on the availability of other water sources and seasonal and interannual climate variability^15^. For this reason, the intention of our GDE map is that it be used as a starting point for prioritizing more refined, localized mapping efforts based on local data and that it be accompanied by verification studies using in situ methods, including local groundwater monitoring. Although it is possible that our random forest model could be modified for localized applications in colder, humid environments, such as by using Sentinel imagery that has a higher temporal frequency than Landsat to avoid cloudy pixels and scan lines in the final map, the application of our random forest map is probably not suitable for subterranean GDEs. Subterranean GDE mapping will require other mapping approaches such as in situ and interference methods based on aquifer mapping. Future work looking at the dynamics of GDEs and fragility would benefit from integrating the perspectives and involvement of local and/or national researchers and practitioners to further refine context-specific interconnections and implications.

### Reporting summary

Further information on research design is available in the Nature Portfolio Reporting Summary linked to this article.

## Online content

Any methods, additional references, Nature Portfolio reporting summaries, source data, extended data, supplementary information, acknowledgements, peer review information; details of author contributions and competing interests; and statements of data and code availability are available at 10.1038/s41586-024-07702-8.

## Supplementary information

Supplementary Information Supplementary Tables 1–3 and 5–6, Figs. 1–3 and references.

Reporting Summary

Supplementary Table 4 Spreadsheet containing ground-truthed vegetation data inventoried within the LFRDB and sPLOTOpen datasets were classified as GDE or non-GDE data according to species and location based on expert and literature review. This table is provided as a separate excel file.

## Figures and Tables

**Extended Data Table 1:**
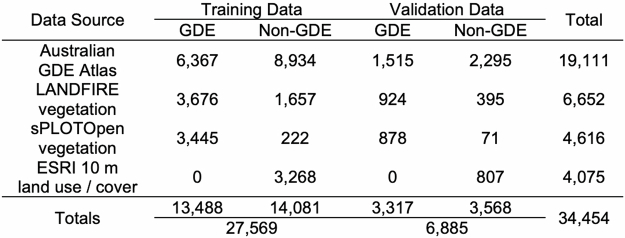
Training and Validation Data used in the Random Forest model

**Extended Data Table 2:**
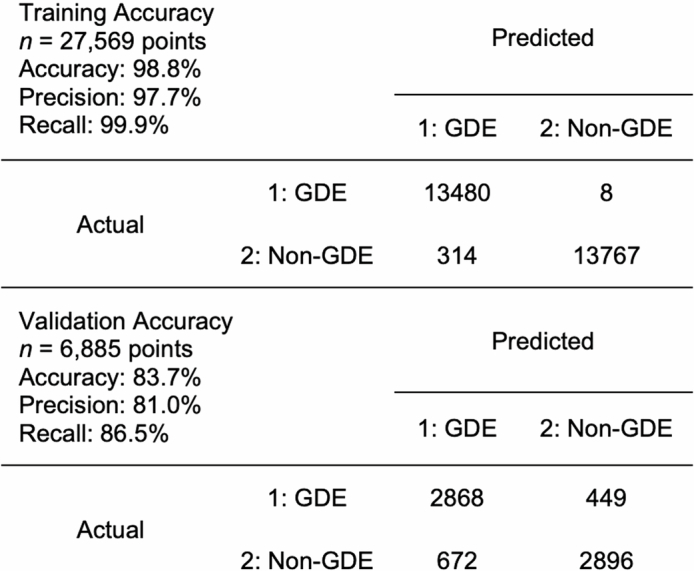
Random forest model confusion matrices based on training (*n* = 27,569 points) and validation (*n* = 6,885 points) data

## Data Availability

GDE data are available at Zenodo (10.5281/zenodo.11062894)^90^. GDE data deposited include the high-resolution (1 arcsecond, roughly 30 m) GDE classification and GDE probability maps, as well as aggregated products of GDE area density at 30 arcsecond (roughly 1 km), 5 arcminute (roughly 10 km) and 30 arcminute (roughly 50 km) resolution. An interactive web map of the high-resolution GDE data is accessible at https://codefornature.projects.earthengine.app/view/global-gde. All source data used in model development and GDE analysis are documented in Supplementary Table 6 and are publicly accessible through the persistent web-links provided.

## Code availability

Code used to generate the global GDE map and produce all results in this study is available alongside the study data at Zenodo (10.5281/zenodo.11062894)^90^. The code repository is also accessible at https://github.com/XanderHuggins/global-gde-map. Code was developed using the R project (v.4.3.1) for statistical computing^91^, Google Earth Engine (https://earthengine.google.com/) and Python (v.3.9.15; https://www.python.org/). R packages necessary for analysis and visualization include terra^92^, rasterDT^93^ and ggplot2 (ref. ^94^). High-resolution global maps were exported using QGIS (http://qgis.org). Composite figures were assembled in Affinity Designer (https://affinity.serif.com/en-us/designer/).
