# Supplementary material for: Groundwater-dependent ecosystem map exposes global dryland protection needs
Source: Nature. 2024 Jul 17;632(8023):101–7. doi: 10.1038/s41586-024-07702-8 (PMC11291274; doi:10.1038/s41586-024-07702-8)
Supplement: Supplementary file 1 — Supplementary Tables 1–3 and 5–6, Figs. 1–3 and references. [file 41586_2024_7702_MOESM1_ESM.pdf]

nature portfolio

https://doi.org/10.1038/s41586-024-07702-8

Supplementary information

Groundwater-dependent ecosystem map exposes global dryland protection needs

In the format provided by the authors and unedited

nature portfolio

https://doi.org/10.1038/s41586-024-07702-8

Supplementary information

Groundwater-dependent ecosystem map exposes global dryland protection needs

In the format provided by the authors and unedited

Groundwater-dependent ecosystem map exposes global dryland protection needs

Melissa M. Rohde[1,2,3*], Christine M. Albano[4], Xander Huggins[5,6,7], Kirk R. Klausmeyer[1], Charles Morton[4], Ali Sharman[8], Esha Zaveri[8], Laurel Saito[9], Zach Freed[10], Jeanette K. Howard[1], Nancy Job[11], Holly Richter[12,13], Kristina Toderich[14,15], Aude-Sophie Rodella[8], Tom Gleeson[5,16], Justin Huntington[4], Hrishikesh A. Chandanpurkar[17,18], Adam J. Purdy[19], James S. Famiglietti[17,20,21], Michael Bliss Singer[22-24*], Dar A. Roberts[25], Kelly Caylor[24-26], John C. Stella[2]

[1] California Water Program, The Nature Conservancy, Sacramento, California, USA
[2] State University of New York, College of Environmental Science and Forestry, Syracuse, New York, USA
[3] Rohde Environmental Consulting, LLC, Seattle, Washington, USA
[4] Division of Hydrologic Sciences, Desert Research Institute, Reno, Nevada, USA
[5] Department of Civil Engineering, University of Victoria, Victoria, Canada
[6] Global Institute for Water Security, University of Saskatchewan, Saskatoon, Canada
[7] International Institute for Applied Systems Analysis, Laxenburg, Austria
[8] The World Bank, Washington, D.C, USA
[9] The Nature Conservancy, Reno, Nevada, USA
[10] Oregon Sustainable Water Program, The Nature Conservancy, Bend, Oregon, USA
[11] Freshwater Biodiversity Programme, South African National Biodiversity Institute, Cape Town, South Africa
[12] The Nature Conservancy, Hereford, Arizona, USA
[13] Resilient Rivers LLC, Hereford, Arizona, USA
[14] International Platform for Dryland Research and Education, Tottori University, Tottori, Japan
[15] Graduate School of Bioresources, Mie University, Tsu, Japan
[16] School of Earth and Ocean Sciences, University of Victoria, Victoria, Canada
[17] Global Institute for Water Security, University of Saskatchewan, Saskatoon, Canada
[18] Center for Sustainability, Environment, and Climate Change, FLAME University, Pune, India
[19] California State University, Monterey Bay, Seaside, California, USA
[20] School of Environment and Sustainability, University of Saskatchewan, Saskatoon, Canada
[21] School of Sustainability, Arizona State University, Tempe, Arizona, USA
[22] School of Earth and Environmental Sciences, Cardiff University, Cardiff, UK
[23] Water Research Institute, Cardiff University, Cardiff, UK
[24] Earth Research Institute, University of California, Santa Barbara, California, USA
[25] Department of Geography, University of California, Santa Barbara, California, USA
[26] Bren School of Environmental Science and Management, University of California, Santa Barbara, Santa Barbara, USA

*Corresponding Authors:
Melissa M. Rohde (melissa@RohdeEnvironmental.com)
Michael Bliss Singer (singerm2@cardiff.ac.uk)

Supplementary Tables and Figures

Supplementary Table 1. Random forest model confusion matrices based on a regional cross-validation test model from the Sahel region (*n* = 4,746 points) using the main model's full training (*n* = 27,569 points) and validation (*n* = 6,885 points) dataset.

Supplementary Table 2. Random forest model confusion matrices based on a regional cross-validation test model that omits training data from Western Australia (*n* = 441 points) from the original training (*n* = 27,110 points) and validation (*n* = 6,903 points).

Supplementary Table 3. Random forest model confusion matrices based on a regional cross-validation test model that omits training data from New Mexico, USA (*n* = 691 points) from the original training (*n* = 26,797 points) and validation (*n* = 6,966 points).

Supplementary Table 4. Ground-truthed vegetation data inventoried within the LANDFIRE 2016 Remap Reference Database (LFRDB) and sPLOTOpen datasets were classified as GDE or non-GDE data according to species and location based on expert and literature review. This table is provided as a separate excel file.

Supplementary Table 5. Vegetation indices used as predictor variables.

Supplementary Table 6. Data sources, descriptions, justifications for inclusion, and preprocessing steps.

Supplementary Fig. 1. Groundwater-dependent ecosystem examples. (a) California, United States: highly fragmented riparian forests amidst an agricultural landscape that supports threatened and endangered species, such as Steelhead (*Oncorhynchus mykiss*) and Least Bell's Vireo (*Vireo bellii pusillus*) (Photo Credit: Melinda Kelley). (b) Rajasthan, India: Shallow groundwater in the Jaisamand Lake Basin supports subsistence farmers and GDEs that are relied on for food and foraging. (Photo Credit: Melissa M. Rohde). (c) Okavango Delta, Botswana: Groundwater and surface water interact to form important seasonal wetlands for migratory animals (Photo Credit: "Botswana Okavango Delta D40_6795" by youngrobv is licensed under CC BY-NC 2.0.). (d) Northern Chad (Sahel): Desert springs support rural livelihoods at Ounianga Serir village next to Lake Teli (Photo Credit: "Ounianga Serir Village" by D-Stanley is licensed under CC BY 2.0). (e) Western Kyzylkum Desert, Uzbekistan: Groundwater-dependent perennial vegetation near Mashikuduk settlement is inhabited by the endangered Grey Monitor (*Varanus griseus* Daudin), the largest lizard in Central Asia (Photo Credit: Kristina Toderich). (f) Salar de Atacama, Bolivia: Shallow brackish groundwater supports important wetlands and flamingos, in one of the world's largest lithium mines (Photo Credit: "Salar de Atacama" by Nicolas de Camaret is licensed under CC BY 2.0).

Supplementary Fig. 2. Hyperparameter tuning for the Western Australia cross-validation test model. Selected hyperparameter values are indicated by the red vertical dashed lines. The red horizontal dashed lines represent the highest accuracy of parameter values.

Supplementary Fig. 3. Hyperparameter tuning for the New Mexico cross-validation test model. Selected hyperparameter values are indicated by the red vertical dashed lines. The red horizontal dashed lines represent the highest accuracy of parameter values.

Supplementary Table 1. Random forest model confusion matrices based on a regional cross-validation test model from the Sahel region (*n* = 4,746 points) using the main model's full training (*n* = 27,569 points) and validation (*n* = 6,885 points) dataset.

**Training Accuracy**<br>*n* = 27,569 points<br>Accuracy: 98.8%<br>Precision: 97.7%<br>Recall: 99.9%		**Predicted**	
		1: GDE	2: Non-GDE
**Actual**	1: GDE	13480	8
	2: Non-GDE	314	13767

**Validation Accuracy**<br>*n* = 6,885 points<br>Accuracy: 83.7%<br>Precision: 81.0%<br>Recall: 86.5%		**Predicted**	
		1: GDE	2: Non-GDE
**Actual**	1: GDE	2868	449
	2: Non-GDE	672	2896

**Sahel Accuracy**<br>*n* = 4,746 points<br>Accuracy: 68.6%<br>Precision: 74.5%<br>Recall: 22.9%		**Predicted**	
		1: GDE	2: Non-GDE
**Actual**	1: GDE	401	1351
	2: Non-GDE	137	2857

Supplementary Table 2. Random forest model confusion matrices based on a regional cross-validation test model that omits training data from Western Australia (*n* = 441 points) from the original training (*n* = 27,110 points) and validation (*n* = 6,903 points).

**Training Accuracy**<br>*n* = 27,110 points<br>Accuracy: 96.2%<br>Precision: 96.6%<br>Recall: 95.5%		**Predicted**	
		1: GDE	2: Non-GDE
**Actual**	1: GDE	12494	588
	2: Non-GDE	440	13588

**Validation Accuracy**<br>*n* = 6,903 points<br>Accuracy: 83.1%<br>Precision: 83.1%<br>Recall: 82.3%		**Predicted**	
		1: GDE	2: Non-GDE
**Actual**	1: GDE	2804	601
	2: Non-GDE	569	2929

**Western Australia Accuracy**<br>*n* = 441 points<br>Accuracy: 53.1%<br>Precision: 79.4%<br>Recall: 47.2%		**Predicted**	
		1: GDE	2: Non-GDE
**Actual**	1: GDE	150	168
	2: Non-GDE	39	84

Supplementary Table 3. Random forest model confusion matrices based on a regional cross-validation test model that omits training data from New Mexico, USA (*n* = 691 points) from the original training (*n* = 26,797 points) and validation (*n* = 6,966 points).

**Training Accuracy**<br>*n* = 26,797 points<br>Accuracy: 99.5%<br>Precision: 98.9%<br>Recall: 100%		**Predicted**	
		1: GDE	2: Non-GDE
**Actual**	1: GDE	13059	0
	2: Non-GDE	147	13591
**Validation Accuracy**<br>*n* = 6,966 points<br>Accuracy: 84.4%<br>Precision: 81.6%<br>Recall: 86.5%		**Predicted**	
		1: GDE	2: Non-GDE
**Actual**	1: GDE	2843	445
	2: Non-GDE	642	3036
**New Mexico Accuracy**<br>*n* = 691 points<br>Accuracy: 61.2%<br>Precision: 68.9%<br>Recall: 75.5%		**Predicted**	
		1: GDE	2: Non-GDE
**Actual**	1: GDE	346	112
	2: Non-GDE	156	77

Supplementary Table 5. Vegetation indices used as predictor variables.

Spectral Index	Equation
**Normalized difference vegetation index (NDVI)**	$NDVI = \frac{NIR - Red}{NIR + Red}$
**Normalized difference Water Index (NDWI)**	$NDWI = \frac{Green - NIR}{Green + NIR}$
**Normalized Difference Moisture Index (NDMI)**	$NDMI = \frac{NIR - SWIR}{NIR + SWIR}$
**Landsat Modified Soil adjusted Vegetation Index (MSAVI)**	$MSAVI = \frac{2NIR + 1 - ((2NIR + 1)^2 - 8(NIR - Red))^{0.5}}{2}$

Supplementary Table 6. Data sources, descriptions, justifications for inclusion, and preprocessing steps.

Dataset	
**Land Cover**	**Data source:** ESRI 10 m Annual Land Use Land Cover (2017-2022)[1]<br>**Persistent web-link:** https://planetarycomputer.microsoft.com/dataset/io-lulc-9-class<br>**Temporal range:** 2017-01-01 to 2022-12-31<br>**Spatial resolution:** 10 m<br>**Description and justification:** High resolution global land use land cover mapping. Pixels mapped as built infrastructure and crops were selected as masks for the model extent, and bare ground pixels were incorporated into the training data.<br>**Preprocessing:** None.
**Annual Mean Global Water Table Depth**<br><br>**"Depth To Groundwater (DTG)"**	**Data source:** Fan et al.[2]<br>**Persistent web-link:** http://thredds-gfnl.usc.es/thredds/catalog/GLOBALWTDFTP/catalog.html<br>**Temporal range:** Mean annual results over a 2004-2013 model run.<br>**Spatial resolution:** 30 arcsecond (~1 km)<br>**Description and justification:** The leading global water table depth dataset. Depth to groundwater (< 30m) was used to define the model extent.<br>**Preprocessing:** Water table depths were smoothed so that surface water features have a depth to groundwater of zero.
**Drylands**	**Data source:** Köppen-Geiger[3]<br>**Persistent web-link:** https://www.gloh2o.org/koppen/<br>**Temporal range:** 1980 - 2016<br>**Spatial resolution:** 1 km<br>**Description and justification:** Leading global climate classification maps that were used to identify global drylands and the model extent.<br>**Preprocessing:** None
**Compound Topographic Index (CTI)**	**Data source:** Marthews et al.[4]<br>**Persistent web-link:** https://catalogue.ceh.ac.uk/documents/6b0c4358-2bf3-4924-aa8f-793d468b92be<br>**Temporal range:** Not applicable.<br>**Spatial resolution:** 15 arc-sec (~ 450 m)<br>**Description and justification:** This global topographic index dataset indicates the propensity of soil to become saturated with water as a result of topographic position. This dataset was used as a predictor variable in the model.

	**Preprocessing:** None
**Annual Precipitation**	**Data source:** TerraClimate[5]<br><br>**Persistent web-link:** https://developers.google.com/earth-engine/datasets/catalog/IDAHO_EPSCOR_TERRACLIMATE#description<br><br>**Temporal range:** 1958-01-01 to 2021-12-01<br><br>**Spatial resolution:** 4638.3 m<br><br>**Description and justification:** Leading climate dataset for global terrestrial surfaces. Annual precipitation data were used to derive a predictor variable in the model.<br><br>**Preprocessing:** None.
**Vegetation Transpiration**	**Data source:** Penman-Monteith-Leuning[6–8]<br><br>**Persistent web-link:** https://developers.google.com/earth-engine/datasets/catalog/CAS_IGSNRR_PML_V2_v017<br><br>**Temporal range:** 2000-02-26 to 2020-12-26<br><br>**Spatial resolution:** 500 m<br><br>**Description and justification:** Leading global vegetation transpiration dataset. Vegetation transpiration data were used to derive a predictor variable in the model.<br><br>**Preprocessing:** None
**Landsat 8 Surface Reflectance Imagery**	**Data source:** USGS<br><br>**Persistent web-link:** https://developers.google.com/earth-engine/datasets/catalog/LANDSAT_LC08_C02_T1_L2<br><br>**Temporal range:** 2013-03-18 to present<br><br>**Spatial resolution:** 30 m<br><br>**Description and justification:** High-resolution global surface reflectance imagery used to generate four vegetation indices and a thermal band that was used to generate ambient land surface temperature spatial anomalies. The vegetation indices and land surface temperature anomaly were all used as predictor variables in the model. While Sentinel satellite data have a higher resolution (10 m), it does not include a thermal band and was too computationally expensive to use in our global model within Google Earth Engine.<br><br>**Preprocessing:** Cloud masking using the 'QA_PIXEL' band, applied scaling factors, and created composite images for the vegetation indices using multi-year (2015-2020) satellite imagery (see code for details).
**LANDFIRE**	**Data source:** LANDFIRE Remap Reference Database (LFRDB)<br><br>**Persistent web-link:** https://landfire.gov/lfrdb.php<br><br>**Temporal range:** 2016<br><br>**Spatial resolution:** Vector data.

	**Description and justification:** Ground-truthed vegetation point data that were used as training data in the model.<br><br>**Preprocessing:** Expert and literature review of species.
**Australian GDE Atlas**	**Data source:** Bureau of Meteorology, Government of Australia<br><br>**Persistent web-link:** http://www.bom.gov.au/water/groundwater/gde/<br><br>**Temporal range:** 2012 to 2019<br><br>**Spatial resolution:** Vector data.<br><br>**Description and justification:** Aquatic and terrestrial GDE data, that are "Known GDE – from regional studies". These data were used as training data in the model.<br><br>**Preprocessing:** None
**sPlotOpen**	**Data source:** Sabatini et al.[9]<br><br>**Persistent web-link:** https://www.idiv.de/en/splot.html<br><br>**Temporal range:** 1888 - 2015<br><br>**Spatial resolution:** 0.01 – 40,000 m^2<br><br>**Description and justification:** Vegetation plot data recording cover or abundance of naturally co-occurring vascular plant species. Species identified as groundwater-dependent were included as training data in the model.<br><br>**Preprocessing:** Expert and literature review of species.
**Groundwater storage trends**	**Data source:** NASA Jet Propulsion Laboratory Level-3 Release 6 Version 2 gridded mascons[10]<br><br>**Persistent web-link:** https://grace.jpl.nasa.gov/data/get-data/<br><br>**Temporal range:** April 2002 to present<br><br>**Spatial resolution:** ½ degree (~56 km at equator)<br><br>**Description and justification:** GRACE-based groundwater storage trends are the most comprehensive data across the globe.<br><br>**Preprocessing:** Groundwater storage anomalies were computed by removing the soil moisture, canopy water storage, and snow water equivalent anomalies from the terrestrial water storage anomalies. The soil moisture, canopy storage, and snow water equivalent time series were obtained from Global Land Data Assimilation System Version 2.1 (GLDAS-2.1) NOAH[11] and VIC (variable infiltration capacity)[12] land surface models.
**Protected Areas**	**Data source:** World Database on Protected Areas (WDPA)<br><br>**Persistent web-link:** https://www.protectedplanet.net<br><br>**Temporal range:** Last accessed June 5, 2023.<br><br>**Spatial resolution:** Vector data.

	**Description and justification:** WDPA is the most comprehensive dataset of protected areas, and were used in the post-hoc analyses.<br><br>**Preprocessing:** We group the WDPA into classes of relatively “high” degrees of protection (WDPA classes Ia, Ib, II, and III), and of relatively “low” degrees of protection (WDPA classes IV, V, and VI).
**Conflict**	**Data source:** Armed Conflict Location & Event Data (ACLED)<br><br>**Persistent web-link:** https://acleddata.com/<br><br>**Temporal range:** January 1, 1997 - Feb. 2, 2021<br><br>**Spatial resolution:** Vector data.<br><br>**Description and justification:** ACLED the leading global dataset on political violence and protests. These data were used in the post-hoc analyses.<br><br>**Preprocessing:** None
**Food Insecurity**	**Data source:** USAID Famine Early Warning Systems Network<br><br>**Persistent web-link:** https://fews.net/data<br><br>**Temporal range:** October 2021<br><br>**Spatial resolution:** District-level vector data.<br><br>**Description and justification:** Leading global dataset of acute food insecurity. These data were used in the post-hoc analyses.<br><br>**Preprocessing:** None
**Pastoral Lands**	**Data source:** Ramankutty et al.[13]<br><br>**Persistent web-link:** https://doi.org/10.1029/2007GB002952<br><br>**Temporal range:** 2000<br><br>**Spatial resolution:** 5 min (~10 km) in longitude by longitude<br><br>**Description and justification:** Derived by agricultural inventory and satellite data. These data were used in the post-hoc analyses.<br><br>**Preprocessing:** None

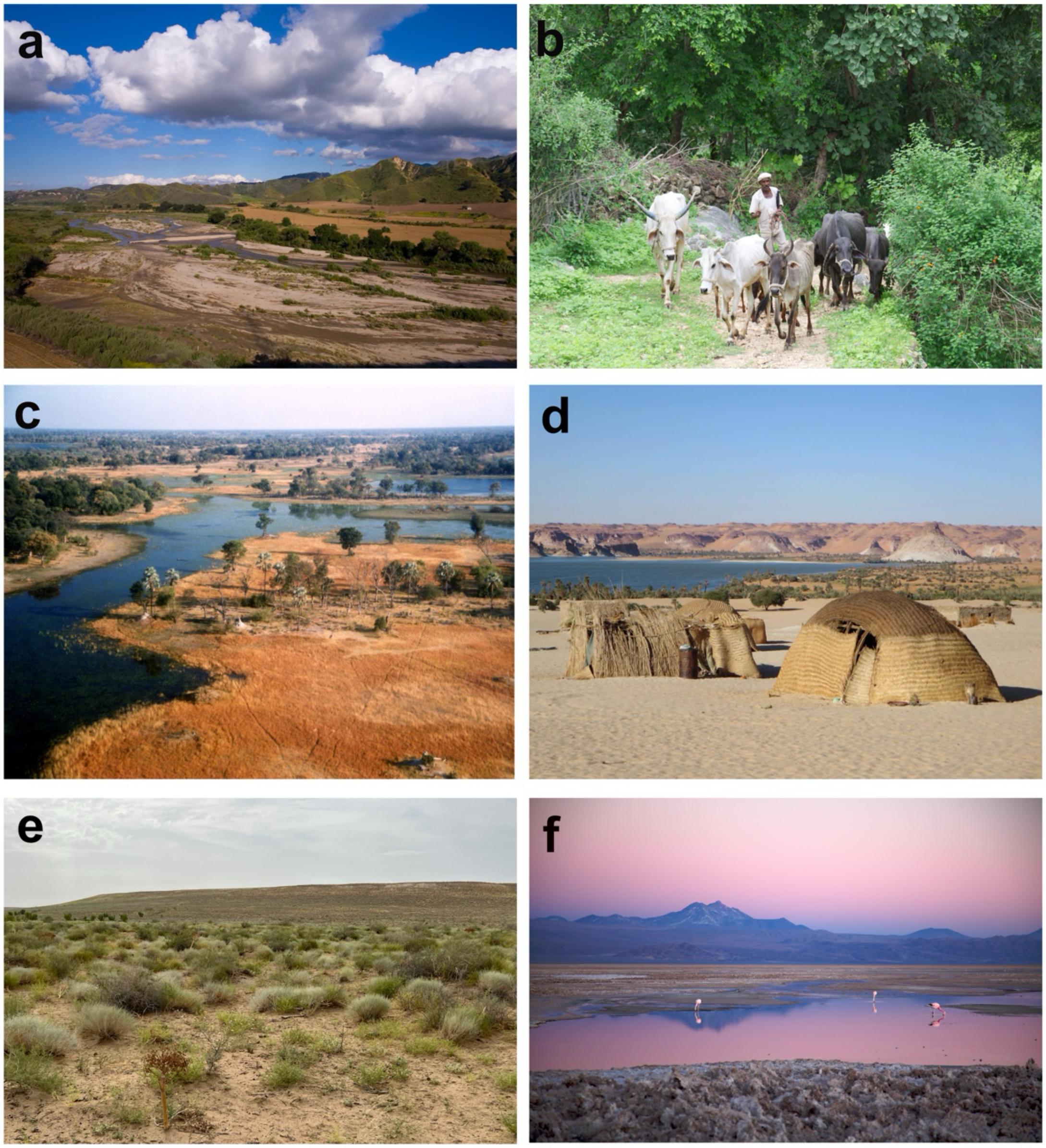

Supplementary Fig. 1. Groundwater-dependent ecosystem examples. (a) California, United States: highly fragmented riparian forests amidst an agricultural landscape that supports threatened and endangered species, such as Steelhead (*Oncorhynchus mykiss*) and Least Bell's Vireo (*Vireo bellii pusillus*) (Photo Credit: Melinda Kelley). (b) Rajasthan, India: Shallow groundwater in the Jaisamand Lake Basin supports subsistence farmers and GDEs that are relied on for food and foraging. (Photo Credit: Melissa M. Rohde). (c) Okavango Delta, Botswana: Groundwater and surface water interact to form important seasonal wetlands for migratory animals (Photo Credit: "Okavango Delta, Botswana" by Carine06 is licensed under CC BY-SA 2.0). (d) Northern Chad (Sahel): Desert springs support rural livelihoods at Ounianga Serir village next to Lake Teli (Photo Credit: "Ounianga Serir Village" by D-Stanley is licensed under CC BY 2.0). (e) Western Kyzylkum Desert, Uzbekistan: Groundwater-dependent perennial vegetation near Mashikuduk settlement is inhabited by the endangered Grey Monitor (*Varanus griseus* Daudin), the largest lizard in Central Asia (Photo Credit: Kristina Toderich). (f) Salar de Atacama, Bolivia: Shallow brackish groundwater supports important wetlands and flamingos, in one of the world's largest lithium mines (Photo Credit: "Salar de Atacama" by Nicolas de Camaret is licensed under CC BY 2.0).

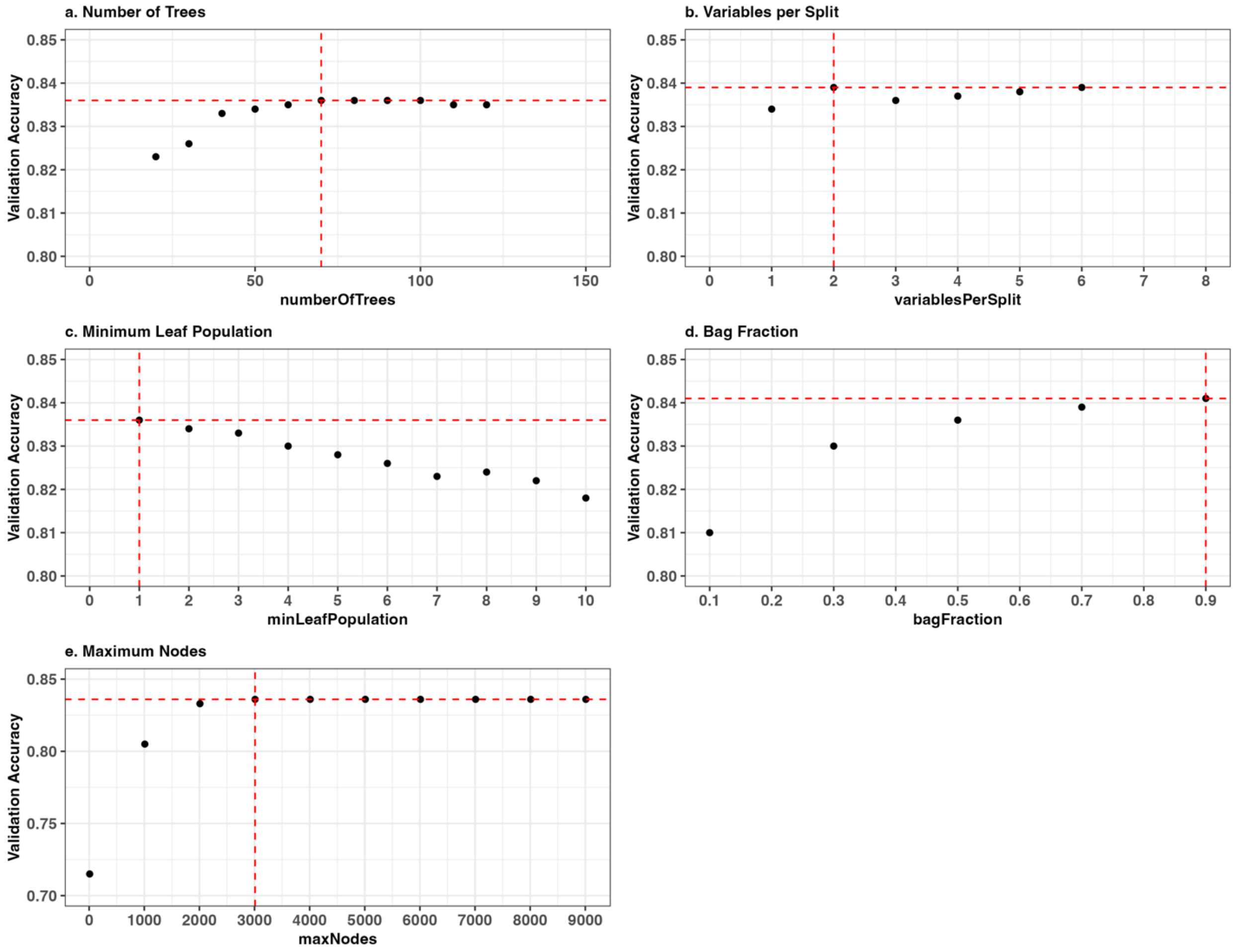

Supplementary Fig. 2. Hyperparameter tuning for the Western Australia cross-validation test model. Selected hyperparameter values are indicated by the red vertical dashed lines. The red horizontal dashed lines represent the highest accuracy of parameter values.

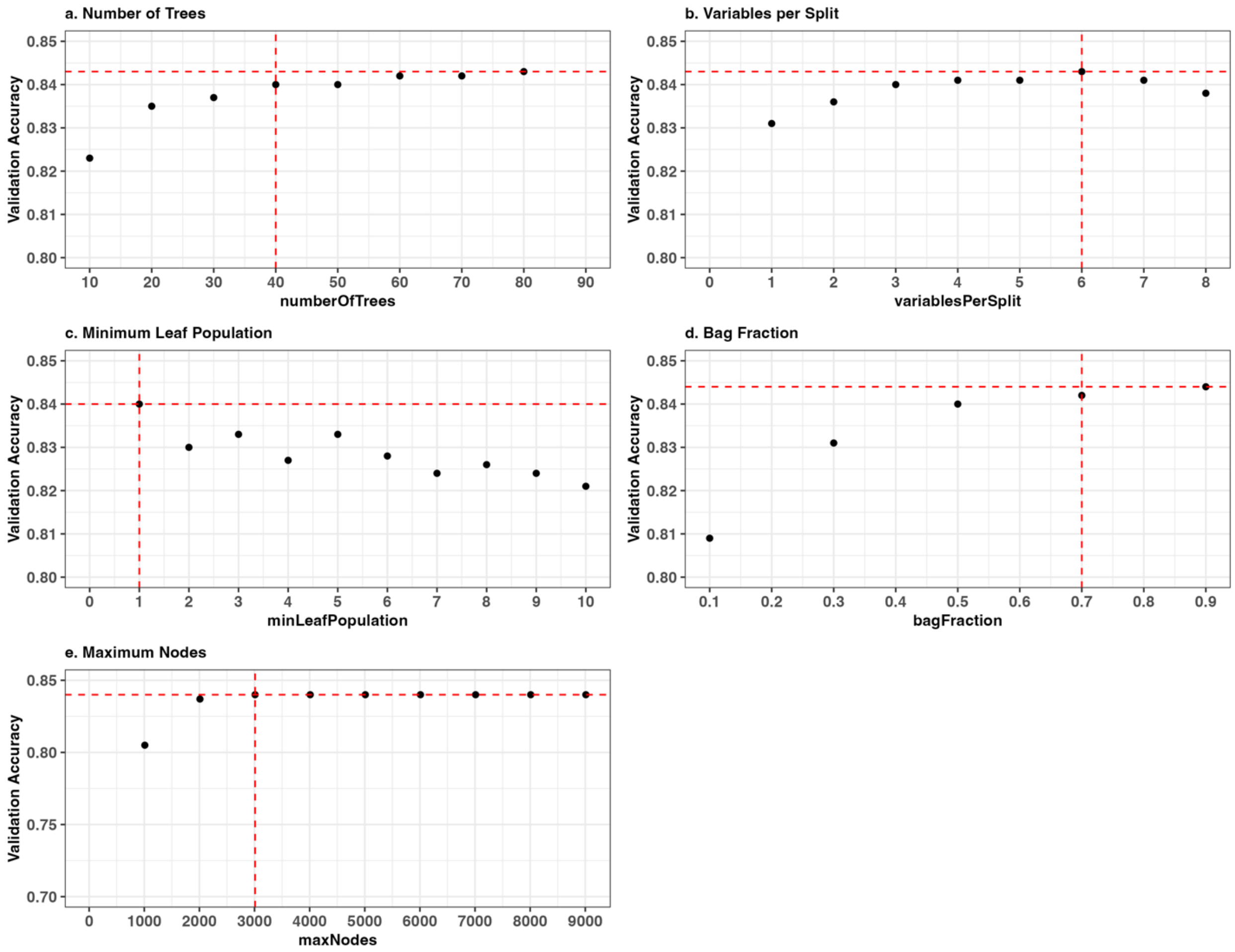

Supplementary Fig. 3. Hyperparameter tuning for the New Mexico cross-validation test model. Selected hyperparameter values are indicated by the red vertical dashed lines. The red horizontal dashed lines represent the highest accuracy of parameter values.

References

1. Karra, K. *et al.* Global land use / land cover with Sentinel 2 and deep learning. in *2021 IEEE International Geoscience and Remote Sensing Symposium IGARSS* 4704–4707 (2021). doi:10.1109/IGARSS47720.2021.9553499.
2. Fan, Y., Miguez-Macho, G., Jobbágy, E. G., Jackson, R. B. & Otero-Casal, C. Hydrologic regulation of plant rooting depth. *Proc National Acad Sci* **114**, 10572–10577 (2017).
3. Beck, H. E. *et al.* Present and future Köppen-Geiger climate classification maps at 1-km resolution. *Sci Data* **5**, 180214 (2018).
4. Marthews, T. R., Dadson, S. J., Lehner, B., Abele, S. & Gedney, N. High-resolution global topographic index values for use in large-scale hydrological modelling. *Hydrology and Earth System Sciences* **19**, 91–104 (2015).
5. Abatzoglou, J. T., Dobrowski, S. Z., Parks, S. A. & Hegewisch, K. C. TerraClimate, a high-resolution global dataset of monthly climate and climatic water balance from 1958–2015. *Sci Data* **5**, 170191 (2018).
6. Zhang, Y. *et al.* Coupled estimation of 500 m and 8-day resolution global evapotranspiration and gross primary production in 2002–2017. *Remote Sensing of Environment* **222**, 165–182 (2019).
7. Gan, R. *et al.* Use of satellite leaf area index estimating evapotranspiration and gross assimilation for Australian ecosystems. *Ecohydrology* **11**, e1974 (2018).
8. Zhang, Y. *et al.* Multi-decadal trends in global terrestrial evapotranspiration and its components. *Sci Rep* **6**, 19124 (2016).
9. Sabatini, F. M. *et al.* sPlotOpen – An environmentally balanced, open-access, global dataset of vegetation plots. *Global Ecology and Biogeography* **30**, 1740–1764 (2021).
10. Watkins, M. M., Wiese, D. N., Yuan, D.-N., Boening, C. & Landerer, F. W. Improved methods for observing Earth's time variable mass distribution with GRACE using spherical cap mascons. *Journal of Geophysical Research: Solid Earth* **120**, 2648–2671 (2015).
11. Ek, M. B. *et al.* Implementation of Noah land surface model advances in the National Centers for Environmental Prediction operational mesoscale Eta model. *Journal of Geophysical Research: Atmospheres* **108**, (2003).
12. Liang, X., Lettenmaier, D. P., Wood, E. F. & Burges, S. J. A simple hydrologically based model of land surface water and energy fluxes for general circulation models. *Journal of Geophysical Research: Atmospheres* **99**, 14415–14428 (1994).
13. Ramankutty, N., Evan, A. T., Monfreda, C. & Foley, J. A. Farming the planet: 1. Geographic distribution of global agricultural lands in the year 2000. *Global Biogeochemical Cycles* **22**, (2008).
