# Supplementary material for: Groundwater-dependent ecosystem map exposes global dryland protection needs
Source: Nature. 2024 Jul 17;632(8023):101–7. doi: 10.1038/s41586-024-07702-8 (PMC11291274; doi:10.1038/s41586-024-07702-8)
Supplement: Supplementary file 2 — Reporting Summary [file 41586_2024_7702_MOESM2_ESM.pdf]

nature portfolio

Corresponding author(s): Melissa M. Rohde and Michael Bliss Singer

Last updated by author(s): July 12, 2024

Reporting Summary

Nature Portfolio wishes to improve the reproducibility of the work that we publish. This form provides structure for consistency and transparency in reporting. For further information on Nature Portfolio policies, see our Editorial Policies and the Editorial Policy Checklist.

Statistics

For all statistical analyses, confirm that the following items are present in the figure legend, table legend, main text, or Methods section.

n/a	Confirmed	
☐	☒	The exact sample size (n) for each experimental group/condition, given as a discrete number and unit of measurement
☐	☒	A statement on whether measurements were taken from distinct samples or whether the same sample was measured repeatedly
☒	☐	The statistical test(s) used AND whether they are one- or two-sided<br>*Only common tests should be described solely by name; describe more complex techniques in the Methods section.*
☒	☐	A description of all covariates tested
☒	☐	A description of any assumptions or corrections, such as tests of normality and adjustment for multiple comparisons
☒	☐	A full description of the statistical parameters including central tendency (e.g. means) or other basic estimates (e.g. regression coefficient) AND variation (e.g. standard deviation) or associated estimates of uncertainty (e.g. confidence intervals)
☒	☐	For null hypothesis testing, the test statistic (e.g. F, t, r) with confidence intervals, effect sizes, degrees of freedom and P value noted<br>*Give P values as exact values whenever suitable.*
☒	☐	For Bayesian analysis, information on the choice of priors and Markov chain Monte Carlo settings
☒	☐	For hierarchical and complex designs, identification of the appropriate level for tests and full reporting of outcomes
☒	☐	Estimates of effect sizes (e.g. Cohen's d, Pearson's r), indicating how they were calculated

Our web collection on statistics for biologists contains articles on many of the points above.

Software and code

Policy information about availability of computer code

Data collection	All data used in this study for model development and analyses are publicly available and can be downloaded from the persistent web-links provided in the Methods section and code (Supplementary Table 6).
Data analysis	Code used to generate the global GDE map and produce all results in this study is deposited alongside the study data in Zenodo (https://doi.org/10.5281/zenodo.11062894). The code repository is also accessible at: https://github.com/XanderHuggins/global-gde-map. Code was developed using the R project for statistical computing (version 4.3.2), Python (version 3.9.15) and Google Earth Engine (https://earthengine.google.com/). R packages necessary for analysis and visualization include terra, rasterDT, and ggplot2. Python modules used include pandas, numpy, and FuzzyWuzzy. High-resolution global maps were exported using QGIS (http://qgis.org). Composite figures were assembled in Affinity Designer (https://affinity.serif.com/en-us/designer/).

For manuscripts utilizing custom algorithms or software that are central to the research but not yet described in published literature, software must be made available to editors and reviewers. We strongly encourage code deposition in a community repository (e.g. GitHub). See the Nature Portfolio guidelines for submitting code & software for further information.

Data

Policy information about availability of data

All manuscripts must include a data availability statement. This statement should provide the following information, where applicable:

- Accession codes, unique identifiers, or web links for publicly available datasets
- A description of any restrictions on data availability
- For clinical datasets or third party data, please ensure that the statement adheres to our policy

The high-resolution global GDE map (1 arcsecond; ~30 m) is available on Zenodo (https://doi.org/10.5281/zenodo.11062894), and the interactive web map is accessible at https://codefornature.projects.earthengine.app/view/global-gde. GDE extent and area density data calculated at 30 arcsecond, 5 arcminute, and 30 arcminute (~1 km, ~10 km, ~ 50km at the equator; respectively) resolutions are also available on the Zenodo data repository. All data used in this study for model development and analyses are publicly available and can be downloaded from the persistent web-links provided in the Methods section and code (Supplementary Table 6).

Research involving human participants, their data, or biological material

Policy information about studies with human participants or human data. See also policy information about sex, gender (identity/presentation), and sexual orientation and race, ethnicity and racism.

Reporting on sex and gender	This information was not collected.
Reporting on race, ethnicity, or other socially relevant groupings	This information was not collected.
Population characteristics	This information was not collected.
Recruitment	This information was not collected.
Ethics oversight	Not applicable.

Note that full information on the approval of the study protocol must also be provided in the manuscript.

Field-specific reporting

Please select the one below that is the best fit for your research. If you are not sure, read the appropriate sections before making your selection.

☐ Life sciences ☐ Behavioural & social sciences ☒ Ecological, evolutionary & environmental sciences

For a reference copy of the document with all sections, see nature.com/documents/nr-reporting-summary-flat.pdf

Ecological, evolutionary & environmental sciences study design

All studies must disclose on these points even when the disclosure is negative.

Study description	Here, we employ a Random Forest machine learning model to provide a high-resolution (1 arcsecond, ~30 m at the equator) spatially explicit global map of probable groundwater-dependent ecosystems (GDEs) in dryland regions. The goals of our map are to: 1) generate a conservative (low) estimate of the likely presence and extent of GDEs; 2) provide a reproducible methodology that allows for periodic mapping to detect changes over time, and which can be refined for regional GDE mapping efforts at various scales using local data and expertise, as well as high-resolution satellite imagery; and 3) serve as a starting point for prioritizing policy and programmatic decisions to enhance GDE monitoring and in-situ validation studies so that GDEs can be protected by relevant groups, organizations, and governments across the globe.
Research sample	Our study is based on publicly-available datasets. See Supplementary Table 6 for a full list of data sources, descriptions, justifications for inclusion, and preprocessing steps.
Sampling strategy	We combine six years (2015-2020) of publicly available datasets (Landsat 8 imagery, climate, topographic, groundwater) and GDE training data (n = 34,454 training points; Extended Data Figure 1 and Extended Data Table 1) to map the likely presence of both aquatic and terrestrial GDEs at ~30 m resolution across global drylands. All data used in this study for model development and analyses are publicly available and can be downloaded from the persistent web-links provided in the Methods section and code (Supplementary Table 6).
Data collection	Our study is based on publicly available datasets. See Supplementary Table 6 for a full list of data sources, descriptions, justifications for inclusion, and preprocessing steps.
Timing and spatial scale	Temporal Scale. Our global groundwater dependent ecosystem (GDE) map was generated using six years (2015-2020) of Landsat imagery. Because our model relies on present-day and available satellite-based thermal and spectral data from the 2015-2020

period, the resultant map reflects the likely location of aquatic and terrestrial GDEs for this snapshot in time.

Spatial Scale. In the absence of a comprehensive global groundwater level database, our Random Forest model utilizes publicly and globally available satellite-based data, including vegetation and water indices, ambient land surface temperature, climate, and topographic data (see Methods). To infer whether ecosystems are being supported by groundwater, our approach assumes that ecosystems with access to groundwater will appear as 'blue or green islands' because they will be wet and maintain ecohydrologic and photosynthetic function during the dry season, in contrast to those without access to groundwater. For this reason, we selected satellite-based data that can measure vegetation greenness, leaf water content, open water bodies, the ratio of the annual sum of actual plant transpiration to precipitation (ETaP), and the spatial anomaly of land surface temperature (LST). ETaP distinguishes pixels in which plant transpiration exceeds precipitation, indicating a likely reliance on groundwater, and LST distinguishes GDEs based on their cooler temperatures relative to the surrounding environment. These cooler temperatures are driven by higher evaporative rates from soil and water bodies influenced by groundwater and higher transpiration rates due to a more abundant water supply available to phreatophytic vegetation. While GDEs exist in both wet and dry environments, the identification of GDEs in humid environments is more difficult using existing satellite-based data because of the inability to differentiate between precipitation and groundwater sources. Thus, we restrict this inference-based approach and the model extent to global drylands Extended Data Figure 2), and exclude places with deep groundwater that are outside the reach of most plant roots (> 30 m; Extended Data Figure 3), in addition to agricultural and urban lands.

Data exclusions	No data were excluded from the analyses.
Reproducibility	All of the results generated in this study are based on publicly-available datasets and are reproducible using our code.
Randomization	Randomization is not relevant to our study since we performed a supervised classification with a Random Forest model to map groundwater dependent ecosystems across global drylands.
Blinding	Blinding is not relevant because in our study we did not perform statistical comparisons between treatment groups in a randomized control trial.

Did the study involve field work? ☐ Yes ☒ No

Reporting for specific materials, systems and methods

We require information from authors about some types of materials, experimental systems and methods used in many studies. Here, indicate whether each material, system or method listed is relevant to your study. If you are not sure if a list item applies to your research, read the appropriate section before selecting a response.

Materials & experimental systems

n/a	Involved in the study	
☒	☐	Antibodies
☒	☐	Eukaryotic cell lines
☒	☐	Palaeontology and archaeology
☒	☐	Animals and other organisms
☒	☐	Clinical data
☒	☐	Dual use research of concern
☒	☐	Plants

Methods

n/a	Involved in the study	
☒	☐	ChIP-seq
☒	☐	Flow cytometry
☒	☐	MRI-based neuroimaging

Plants

Seed stocks	Not applicable.
Novel plant genotypes	Not applicable.
Authentication	Not applicable.
